# Nomilin Rewires mTOR-Driven Immunometabolism via DDIT4 to Restrain Psoriasis

**DOI:** 10.7150/ijbs.133330

**Published:** 2026-09-01

**Authors:** Ha Eun Kim, Seung Taek Lee, Hee-Suk Park, Hyun-Su Lee, Jong Yeong Lee, Dae-Ki Kim, Si-Gyun Roh, Kyung-Hwa Nam, Sang-Hyun Kim, Jin Kyeong Choi

**Affiliations:** 1 Department of Immunology, Jeonbuk National University Medical School, Jeonju, 54907, Republic of Korea.; 2 Department of Physiology, Daegu Catholic University School of Medicine, Daegu, 42472, Republic of Korea.; 3 Department of Plastic and Reconstructive Surgery, Jeonbuk National University Medical School, Jeonju, Republic of Korea; Biomedical Research Institute of Jeonbuk National University Hospital, Institute for Medical Sciences, Jeonbuk National University, Jeonju, 54907, Republic of Korea.; 4 Department of Dermatology, Jeonbuk National University Medical School, Jeonju, Republic of Korea; Biomedical Research Institute of Jeonbuk National University Hospital, Institute for Medical Sciences, Jeonbuk National University, Jeonju, 54907, Republic of Korea.; 5 CMRI, Department of Pharmacology, School of Medicine, Kyungpook National University, Daegu, 41944, Republic of Korea.; 6 Biomedical Research Institute of Jeonbuk National University Hospital, Institute for Medical Sciences, Jeonbuk National University, Jeonju, 54907, Republic of Korea.

**Keywords:** nomilin, psoriasis, immunometabolism, mTOR, DDIT4, mitochondrial ROS

## Abstract

Nomilin, a citrus-derived limonoid belonging to the tetranortriterpenoid family, has been reported to exert anti-inflammatory and antioxidant effects; however, its therapeutic relevance and underlying mechanisms in psoriasis remain unclear. Here, we investigated the effects of nomilin in 12-O-tetradecanoylphorbol-13-acetate (TPA)- and imiquimod (IMQ)-induced psoriasis-like mouse models, IL-17A-stimulated primary human keratinocytes, Th17-polarized CD4⁺ T cells, and peripheral blood mononuclear cells (PBMCs) from patients with psoriasis. Nomilin markedly alleviated epidermal hyperplasia and inflammatory symptoms, suppressed pro-inflammatory cytokines and psoriasis-associated mediators, and reduced the expansion of inflammatory myeloid and Th1/Th17 cells in lesional skin. Mechanistically, nomilin decreased glucose uptake, glycolytic activity, oxidative phosphorylation, mitochondrial mass, and mitochondrial ROS production, thereby preventing metabolic overactivation and mitochondrial dysfunction. At the molecular level, nomilin upregulated DDIT4 and inhibited mTOR signaling, as evidenced by reduced phosphorylation of mTOR, RAPTOR, p70S6K, and 4EBP1. Genetic silencing of DDIT4 abrogated the anti-inflammatory and metabolic effects of nomilin in keratinocytes, while skin-specific DDIT4 knockdown markedly attenuated its therapeutic efficacy *in vivo*, demonstrating an essential role for DDIT4 in nomilin-mediated mTOR suppression. Furthermore, molecular docking and cellular thermal shift assay (CETSA) analyses revealed a direct interaction between nomilin and DDIT4. In addition, nomilin suppressed Th17 differentiation and reduced TNF-α-, IFN-γ-, and IL-17A-producing CD4⁺ T cells in PBMCs from patients with psoriasis. Collectively, these findings identify nomilin as a modulator of the DDIT4-mTOR axis that alleviates psoriasis-like inflammation through the rewiring of pathogenic immunometabolic programs and highlight its potential as a therapeutic strategy for psoriasis.

## Introduction

Psoriasis is a chronic immune-mediated inflammatory skin disorder characterized by epidermal hyperplasia, vascular remodeling, and infiltration of activated leukocytes 1, 2. Aberrant crosstalk between innate and adaptive immune compartments sustains a pathogenic cytokine network dominated by TNF-α, IFN-γ, and IL-17, which drives keratinocyte hyperproliferation and chronic tissue remodeling 3-7. Although biologics targeting IL-17 or TNF-α have improved clinical outcomes, long-term immune suppression is associated with safety concerns and incomplete disease resolution, highlighting the need for strategies that restore immune and metabolic homeostasis rather than broadly blocking cytokine signaling 3-8.

Emerging evidence indicates that psoriatic inflammation is tightly coupled to metabolic reprogramming 8-10. Both keratinocytes and infiltrating immune cells exhibit enhanced glucose uptake, increased glycolytic flux, and elevated oxidative phosphorylation (OXPHOS), supporting sustained cytokine production and proliferative expansion 11-13. Importantly, mitochondrial dysfunction and excessive reactive oxygen species (ROS) generation further amplify inflammatory signaling, suggesting that metabolic activation is not merely a downstream consequence of inflammation but a mechanistic driver of disease progression 8, 14.

Mechanistic target of rapamycin (mTOR) integrates nutrient availability and inflammatory signals to coordinate cell growth, differentiation and metabolic programming 15, 16. In psoriatic lesions, mTORC1 activity promotes Th17 differentiation and keratinocyte hyperactivation, and pharmacologic inhibition of mTOR attenuates experimental disease severity 8, 17, 18. These observations position mTOR-driven metabolic activation as a central regulatory node in psoriasis pathogenesis.

DNA damage-inducible transcript 4 (DDIT4, also known as REDD1) 19 is a stress-responsive inhibitor of mTORC1 that is induced by hypoxia, oxidative stress and metabolic imbalance 20-24. Through negative regulation of mTOR signaling, DDIT4 modulates mitochondrial function and metabolic adaptation 21, 25, 26. Although DDIT4 has been implicated in inflammatory and metabolic stress responses 26, 27; however, whether DDIT4 serves as a regulatory node linking metabolic reprogramming to pathogenic immune activation in psoriasis remains largely unknown.

Nomilin is a citrus-derived limonoid that has attracted considerable attention because of its broad pharmacological activities. Previous studies have demonstrated that nomilin exerts anti-inflammatory and immunomodulatory effects through suppression of NF-κB, MAPK, and NFATc1 signaling pathways, resulting in reduced inflammatory cytokine production and immune cell activation 28-32. In addition, nomilin has been shown to attenuate obesity-associated metabolic dysfunction and hyperglycemia by regulating energy homeostasis 33. Nomilin also exhibits antioxidant and cytoprotective activities through activation of the Nrf2 pathway, thereby reducing oxidative stress and tissue injury 34. Furthermore, nomilin enhances phase II detoxification responses, including glutathione-S-transferase activity, and has been reported to modulate mitochondrial and cellular metabolic functions 31, 35. Although the role of nomilin in psoriasis has not previously been investigated, these established biological activities provided a strong rationale for evaluating its therapeutic potential in psoriasis. Given that psoriasis is increasingly recognized as an immunometabolic disorder characterized by aberrant mTOR activation, metabolic rewiring, mitochondrial dysfunction, oxidative stress, and pathogenic Th17 responses, we hypothesized that nomilin may attenuate psoriatic inflammation by targeting disease-associated immunometabolic activation. However, whether nomilin regulates DDIT4-dependent mTOR signaling and metabolic reprogramming in psoriasis remains unknown.

Here, we demonstrate that nomilin attenuates psoriasiform skin inflammation by suppressing mTORC1-dependent metabolic activation. Using TPA- and IMQ-induced murine models, IL-17A-stimulated primary human keratinocytes, Th17-polarized CD4⁺ T cells, and patient-derived PBMCs, we show that nomilin suppresses glucose metabolism, mitochondrial ROS production, and pathogenic effector T-cell responses. Mechanistically, these effects require DDIT4 and are associated with inhibition of mTORC1 signaling. Together, our findings identify nomilin as a metabolic regulator that selectively restrains pathogenic immune activation in psoriasis.

## Materials and Methods

### Ethical statement

This study was approved by the Institutional Review Board (IRB) of Jeonbuk National University Hospital (Approval No. 2022-05-002-012). All human blood and tissue samples were obtained from patients after written informed consent was secured prior to specimen collection. The study was conducted in accordance with the principles of the Declaration of Helsinki for biomedical research involving human subjects. All animal experiments were reviewed and approved by the Institutional Animal Care and Use Committee (IACUC) of Jeonbuk National University (Approval No. JBNU NON2024-163-001). All procedures were performed in accordance with the institutional guidelines and national regulations for the care and use of laboratory animals.

### Human samples

Peripheral blood samples were obtained from patients with plaque psoriasis treated at Jeonbuk National University Hospital. A total of 21 patients with psoriasis were enrolled in this study. Clinical characteristics, including age, sex, PASI score, and disease severity, are summarized in Supplementary Table S1. Disease severity was assessed using the Psoriasis Area and Severity Index (PASI). Eligible participants were adults aged 18-70 years with clinically diagnosed plaque psoriasis of at least 3 months' duration. Patients with active infection, other autoimmune or systemic diseases, or recent use of systemic immunosuppressive or biologic therapies were excluded. All participants provided written informed consent prior to sample collection.

### Animals

Eight-week-old female C57BL/6J mice were purchased from Nara Biotech (Pyeongtaek-si, Gyeonggi-do, Republic of Korea). The weight of the mice ranged from 18-21g. Animals were housed under controlled conditions (20 ± 5°C; relative humidity 50 ± 5%) with free access to food and water. A 12 h light/dark cycle was maintained with automatic lighting control.

### IMQ-induced psoriasis-like mouse model

Psoriasis-like skin inflammation was IMQ-induced as previously described 9 by daily topical application of 62.5 mg of 5% imiquimod cream (Aldara; Dong-A Pharmaceutical Co., Korea) to the shaved dorsal skin and both ears of mice for 8 consecutive days (days 0-7). Nomilin (Sigma-Aldrich, St. Louis, MO) was administered intraperitoneally at low (0.2 mg/mouse) and high (0.4 mg/mouse) doses, while rapamycin (0.6 mg/mouse;MedChemExpress, Monmouth Junction, NJ) was used as a positive control. All treatments were administered once daily from day 1 to day 7. Clinical severity was assessed daily based on erythema, scaling, and skin thickening scores. Ear thickness was measured daily using a digital thickness gauge (Mitutoyo Co., Tokyo, Japan), and dorsal skin thickness was determined on the day of sacrifice. On day 8, mice were euthanized by CO₂ inhalation, and dorsal skin and ear tissues were collected. Tissue samples were either fixed in 4% formaldehyde for histological analysis or stored at -80°C for subsequent qPCR and Western blot analyses. Additional tissue samples were processed for flow cytometric analysis, Seahorse metabolic assays, and MitoTracker staining.

### IMQ-induced psoriasis-like ear inflammation model with siDDIT4 silencing

An IMQ-induced psoriasis-like ear inflammation model was established as previously described 36. Mice were randomly assigned to the indicated experimental groups (*n* = 4 mice per group). To investigate the role of DDIT4 in the therapeutic effects of nomilin, mice received daily intradermal injections of Accell™ SMART pool siDDIT4 (5 μM; Horizon Discovery, Cambridge, UK), a self-delivering siRNA platform optimized for *in vivo* gene silencing without the need for transfection reagents 37, into both ears together with intraperitoneal administration of nomilin (0.4 mg/mouse) from day 1 to day 7. Ear thickness was measured daily using a digital thickness gauge (Mitutoyo Co., Tokyo, Japan). On day 8, mice were euthanized, and ear tissues were collected for histological, qPCR, and Western blot analyses.

### Histological analysis

Ear and dorsal tissues were fixed in 4% formalin and embedded in paraffin. Sections with a thickness of 4 μm were stained with hematoxylin and eosin. Epidermal and dermal thicknesses were measured from H&E-stained sections using images acquired with a 20× objective lens on an APX100 All-in-One Fluorescence Microscope (Evident, Tokyo, Japan). Measurements were obtained from five randomly selected fields per sample.

### Quantitative real-time PCR

Total RNA was extracted from mouse ear tissues and cultured primary human keratinocytes using RNAiso Plus reagent (Takara, Tokyo, Japan). RNA was purified by phenol/chloroform extraction, washed with 75% DEPC-treated ethanol, and resuspended in RNase-free water. RNA concentration and purity were assessed using a NanoDrop spectrophotometer (Thermo Fisher Scientific). cDNA was synthesized using the RevertAid First Strand cDNA Synthesis Kit (Thermo Fisher Scientific). Quantitative real-time PCR (qPCR) was performed using SYBR Green Master Mix (NanoHelix, Daejeon, Korea) on a StepOnePlus Real-Time PCR System (Thermo Fisher Scientific). Relative gene expression levels were calculated using the comparative ΔΔCt method and normalized to β-actin as an internal reference gene. The primer sequences used for qPCR are listed in Supplementary Table S2.

### Western blot

Proteins were extracted from primary human keratinocytes, human peripheral blood mononuclear cells (PBMCs), mouse Th17 cells, and cells isolated from mouse ear and dorsal skin tissues. Western blot analysis was performed as described previously, and proteins were separated according to molecular weight using SDS-PAGE (6-15%). Protein expression was detected using specific primary antibodies, and immunoblotting was performed following standard protocols. Detailed information regarding all primary and secondary antibodies used for Western blot analysis, including catalog numbers, suppliers, and working dilutions, is provided in Supplementary Table S3. After incubation with the corresponding secondary antibodies, protein bands were visualized using ECL Select Western Blotting Detection Reagent (Cytiva Life Sciences, Marlborough, MA, USA; Cat#RPN2235). Chemiluminescent signals were detected and quantified using an Amersham Imager 600 (Cytiva Life Sciences, Marlborough, MA, USA). Band intensities were quantified using ImageJ software (National Institutes of Health, Bethesda, MD, USA).

### Flow cytometry

On the day of sacrifice, skin tissues, including ear and dorsal skin samples, were collected, minced, and cultured in a medium containing Liberase^TM^ TH Research Grade (Roche Diagnostics, Mannheim, Germany; Cat#5401135001) for 1 h 30 min. The cell suspension was filtered through a 100 μm nylon cell strainer, centrifuged at 400 × g for 5 min, and subsequently passed through a 40 μm nylon cell strainer, followed by an additional centrifugation at 400 × g for 5 min. Red blood cells were removed using ammonium chloride-potassium (ACK) lysis buffer, and the cells were washed with PBS. For intracellular cytokine detection, cells were stimulated with phorbol 12-myristate 13-acetate (PMA; 20 ng/mL; Sigma-Aldrich, St. Louis, MO, USA; Cat#P1585), ionomycin (1 μg/mL; Sigma-Aldrich, St. Louis, MO, USA; Cat#I0634), and Golgi Plug (BD Biosciences, San Jose, CA, USA; Cat#555029) for 4 h. After stimulation, cells were washed twice with PBS. Fixable Viability Dye eFluor 780 (Invitrogen, Carlsbad, USA; APC-Cy7; Cat#65-0865-14) was applied for 15 min at 4°C, followed by two washes with cold PBS. For surface staining, cells were incubated with fluorochrome-conjugated antibodies for 20 min at 4°C. T-cell populations were stained with anti-mouse CD4 and anti-mouse CD25 antibodies. Myeloid cell populations were stained with anti-mouse CD11b, anti-mouse F4/80, anti-mouse Ly6C, anti-mouse Ly6G, and anti-mouse CD86 antibodies. Following surface staining, cells were fixed and permeabilized using the Foxp3/Transcription Factor Fixation/Permeabilization Kit according to the manufacturer's instructions. Intracellular staining was subsequently performed overnight at 4°C using anti-mouse IFN-γ, anti-mouse IL-17A, anti-mouse IL-22, anti-mouse Foxp3, anti-mouse RORγt, and anti-mouse CD163 antibodies. Detailed information regarding fluorochromes, clones, catalog numbers, and suppliers for all antibodies is provided in Supplementary Table S4. Samples were acquired using an Attune NxT Acoustic Focusing Cytometer (Thermo Fisher Scientific, Waltham, MA, USA) and analyzed using FlowJo software (version 10.7.1).

### Glucose uptake assay

Primary human keratinocytes (5 × 10^5^ cells/well) were plated in KSFM medium and seeded into 96 well Flat-bottom plates. Mouse ear and dorsal skin tissue cells (5 × 10^5^ cells/well) were plated in complete RPMI and seeded into 96 well U-bottom plates. Cells were incubated with 2-[N-(7-nitrobenz-2-oxa-1,3-diazol-4-yl) amino]-2-deoxy-D-glucose (2-NBDG, 0.01 mg/mL; Thermo Fisher Scientific, Waltham, MA, USA; Cat#N13195) for 30 min at 37°C in the dark. After incubation, cells were washed once with PBS and subsequently stained with Live/Dead Fixable Dead Cell Stain for 20 min at room temperature, protected from light, to distinguish live and dead cells. Samples were analyzed using an Attune NxT Acoustic Focusing Cytometer.

### Seahorse metabolic analysis

Skin single cells isolated from mouse ear tissues were seeded at a density of 1 × 10^6^ cells/well onto poly-D-lysine (Gibco)-coated Seahorse XFp cell culture miniplates (Agilent Technologies, Santa Clara, USA; Cat#103576-100). Cells were incubated in Seahorse XF RPMI assay medium supplemented with 10 mM glucose, 2 mM glutamine, and 1 mM pyruvate and equilibrated at 37°C in a non-CO₂ incubator prior to analysis. OCR was measured using the Seahorse XFp Cell Mito Stress Test Kit (Agilent Technologies, Santa Clara, USA; Cat#103015-100) according to the manufacturer's instructions. Sequential injections of oligomycin, FCCP, and rotenone/antimycin A were performed to assess basal respiration, maximal respiration, and spare respiratory capacity. ECAR was determined using the Seahorse XF Glycolysis Stress Test Kit (Agilent Technologies, Santa Clara, USA; Cat#103020-100). Following glucose starvation in XF assay medium, sequential injections of glucose, oligomycin, and 2-deoxy-D-glucose (2-DG) were performed to evaluate glycolysis, glycolytic capacity, and glycolytic reserve. OCR and ECAR values were normalized to cell number prior to analysis to ensure comparability across experimental groups. All measurements were acquired using a Seahorse XFp Analyzer (Agilent Technologies, Santa Clara, USA) and analyzed with Wave software (Agilent Technologies, Santa Clara, USA).

### Primary human keratinocyte stimulation

For stimulation experiments, primary human keratinocytes were exposed to recombinant human IL-17A (200 ng/mL; PeproTech, Rocky Hill, NJ, USA; Cat#200-17) for 24 h, either alone or in combination with Nomilin (50 μg/mL; Sigma-Aldrich, St. Louis, MO, USA; Cat#PHL80507). Where indicated, Rapamycin (100 nM; GenDEPOT, Katy, TX, USA; Cat#BC082-050) was co-administered for comparative analyses.

### siRNA transfection

Primary human keratinocytes were seeded at approximately 70% confluency and allowed to stabilize in complete medium. Prior to transfection, the medium was replaced with serum-free medium. Lyophilized siRNA targeting DDIT4/REDD1 was reconstituted in siRNA Dilution Buffer (Santa Cruz Biotechnology, Dallas, TX, USA) to obtain a 10 μM stock solution. A non-targeting control siRNA (siControl; Santa Cruz Biotechnology, Dallas, TX, USA; Cat#sc-37007) and DDIT4/REDD1 siRNA (siDDIT4; Santa Cruz Biotechnology, Dallas, TX, USA; Cat#sc-45806) and AMPKα siRNA (siAMPK; Santa Cruz Biotechnology, Dallas, TX, USA; Cat#sc-45312) were used. Transfection complexes were prepared by mixing Fugene reagent with Transfection Medium (Santa Cruz Biotechnology, Dallas, TX, USA; Cat#sc-36868) and siRNA according to the manufacturer's instructions. The mixtures were gently vortexed and incubated for 30 min at room temperature to allow complex formation. The complexes were then added dropwise to the cells at final working volumes of 10 μL per well for 96 well plates (in a total medium volume of 100 μL), 50 μL per well for 24 well plates (in a total medium volume of 500 μL), or 250 μL per well for 6 well plates (in a total medium volume of 1 mL). Cells were incubated under standard culture conditions for 48 h to achieve DDIT4 knockdown. Cells transfected with non-targeting siRNA served as the negative control. Following transfection, cells were washed once with pre-warmed PBS, and the medium was replaced with complete keratinocyte serum-free medium (KSFM). Stimulatory reagents were subsequently added according to the experimental design.

### Immunofluorescence staining and confocal microscopy

Primary human keratinocytes were transfected with siControl or siDDIT4 for 48 h prior to stimulation. Cells (1 × 10^6^ cells/well) were seeded onto collagen-coated Millicell EZ Slide 8 well glass chamber slides (MilliporeSigma, Burlington, MA, USA; Cat#PEZGS0816) slides and treated with IL-17A (200 ng/mL) in the presence or absence of Nomilin (50 μg/mL) for 24 h. Following stimulation, cells were incubated with MitoTracker Green FM (0.2 μM; Thermo Fisher Scientific, Waltham, MA, USA; M7514), MitoTracker Red CMXRos (0.2 μM; Thermo Fisher Scientific, Waltham, MA, USA; Cat#M46752), and MitoSOX Red Mitochondrial Superoxide Indicator (1 μM; Thermo Fisher Scientific, Waltham, MA, USA; Cat#M36008) at 37°C for 60 min in the dark. After staining, cells were washed once with 1× PBS and fixed with 4% paraformaldehyde for 15 min at room temperature. Cells were permeabilized with 0.3% Triton X-100 for 10 min and counterstained with DAPI (Thermo Fisher Scientific, Waltham, MA, USA; Cat#62248) for nuclear visualization. Coverslips were mounted using fluorescent mounting medium, and images were acquired using a ZEISS confocal laser scanning microscope at 63× objective under identical acquisition settings for all groups. Fluorescence intensity was quantified using ImageJ software. Relative fluorescence intensity was normalized to the unstimulated group. Scale bars represent 10 μm.

### Molecular docking analysis

Molecular docking analysis was performed to evaluate the binding interactions of nomilin with DDIT4 and the mTOR-FKBP12 complex, as well as the interaction of rapamycin with the mTOR-FKBP12 complex. Docking simulations were performed using AutoDock Vina with default parameters. Grid boxes were defined to encompass the corresponding ligand-binding regions, and binding affinities were calculated in kcal/mol.

### Statistical analysis

All experiments were performed at least three independent times unless otherwise indicated. Data are presented as mean ± SEM. Statistical analyses were conducted using GraphPad Prism software (version 9.0; GraphPad Software, San Diego, CA, USA). For comparisons between two groups, an unpaired two-tailed Student's t-test was used. For comparisons among multiple groups, one-way ANOVA followed by Holm-Šídák multiple comparison test was performed. Time-course data were analyzed using two-way ANOVA followed by Holm-Šídák post hoc test, as appropriate. A p value < 0.05 was considered statistically significant. Statistical significance was defined as follows: p < 0.05 (*), p < 0.01 (**), p < 0.001 (***), and p < 0.0001 (****).

## Results

### Nomilin attenuates experimental psoriasiform skin inflammation *in vivo*

Topical application of 12-O-tetradecanoylphorbol-13-acetate (TPA), a protein kinase C activator, induces acute epidermal hyperplasia and inflammatory cytokine production in murine skin and is widely used to model psoriasiform inflammation 38-41. The molecular structure of the citrus triterpenoid nomilin is shown in Fig. 1A. To evaluate its anti-inflammatory efficacy, C57BL/6 mice received daily topical TPA on the ear for 7 days, with concurrent administration of nomilin (200 or 400 μg per mouse) or the mTOR inhibitor rapamycin (600 μg per mouse) as outlined in Fig. 1B. Body weight remained stable throughout the induction period across all groups (Fig. 1C). TPA-treated ears developed marked erythema, scaling, and thickening compared with vehicle controls (Fig. 1D). Both doses of nomilin reduced these visible inflammatory features. Consistent with these observations, TPA treatment resulted in a progressive increase in ear thickness over time (Fig. 1E), which was significantly attenuated by nomilin at both doses, without a clear dose-dependent difference. Clinical severity was further evaluated using a standardized 0-4 scoring system assessing scaling, thickness, and erythema. TPA markedly increased all three parameters. Both rapamycin and nomilin improved the clinical severity scores, with nomilin showing greater efficacy (Fig. 1F). Histological examination corroborated these findings (Fig. 1D). TPA markedly increased epidermal and dermal thickness. Both nomilin and rapamycin significantly reduced epidermal and dermal thickness parameters (Fig. 1G). Nomilin also significantly reduced TPA-induced enlargement of draining lymph nodes and spleen (Supplementary Fig. S1A). TPA robustly upregulated the mRNA expression of pro-inflammatory cytokine expression, including *Tnfα* and *Il1β*, as well as psoriasis-associated mediators (*Lcn2*, *S100a7*, *S100a8*, and *S100a9*), all of which were significantly suppressed by nomilin treatment (Supplementary Fig. S1B).

To further validate these findings in a clinically relevant model, we next employed the imiquimod (IMQ)-induced psoriasis-like skin inflammation model (Fig. 1H), which activates the IL-23/IL-17 axis and recapitulates key immunopathological features of human psoriasis, including Th17-driven inflammation and epidermal hyperplasia 42. Similar to the TPA model, body weight remained unchanged throughout the experimental period (Fig. 1I). IMQ application induced marked erythema, scaling, skin thickening, and epidermal hyperplasia, whereas nomilin treatment substantially ameliorated these pathological features (Fig. 1J-M). Nomilin significantly reduced dorsal skin thickness and clinical severity scores, including scaling, erythema, and thickness (Fig. 1J-L). Histological analysis further confirmed significant reductions in epidermal and dermal thickness in nomilin-treated mice compared with IMQ controls (Fig. 1M). In addition, nomilin significantly reduced IMQ-induced enlargement of the spleen and draining lymph nodes (Supplementary Fig. S1C) and suppressed the mRNA expression of inflammatory cytokines and psoriasis-associated mediators, including *Tnfα*, *Il1β*, *Lcn2*, *S100a7*, *S100a8*, and *S100a9* (Supplementary Fig. S1D). Collectively, these findings demonstrate that nomilin effectively attenuates psoriasiform skin inflammation in both TPA- and IMQ-induced mouse models.

### Nomilin suppresses myeloid and T cell-mediated inflammatory responses in psoriasis-like lesions

To assess whether nomilin modulates immune cell infiltration within psoriasis-like lesions, we performed flow cytometric analysis of skin-infiltrating leukocytes isolated from IMQ-induced psoriasiform lesions (Fig. 2A). Representative contour plots of neutrophils and macrophages are shown in Fig. 2B, with corresponding quantification in Fig. 2C. IMQ stimulation significantly increased the frequencies of CD11b⁺Ly6G⁺ neutrophils and CD11b⁺F4/80⁺ macrophages compared with control mice. Nomilin treatment markedly reduced the accumulation of both cell populations in lesional skin. Rapamycin also reduced the infiltration of both neutrophils and macrophages; however, nomilin more effectively suppressed the infiltration of each cell population (Fig. 2B). CD4⁺ T-cell subsets were further analyzed. Compared with controls, IMQ stimulation induced a significant expansion of Th1 (CD4⁺IFN-γ⁺), Th17 (CD4⁺IL-17A⁺), and Th22 (CD4⁺IL-22⁺) cell populations. Nomilin administration significantly suppressed all three pathogenic effector T-cell subsets (Fig. 2C). In contrast, the frequency of CD4⁺CD25⁺Foxp3⁺ regulatory T cells was significantly increased following nomilin treatment, indicating a shift toward an immunoregulatory phenotype. Rapamycin exerted comparable effects on most T-cell populations, although subtle differences were observed in selected subsets.

Similar immune alterations were observed in the TPA-induced model. The TPA-induced increase in neutrophil and macrophage frequencies was significantly attenuated by nomilin in both lesional skin and draining lymph nodes (Supplementary Fig. S2A-C). In addition, nomilin suppressed the expansion of Th1- Th17- and Th22-associated cell populations while increasing Foxp3⁺ regulatory T cells in draining lymph nodes (Supplementary Fig. S2D-E), indicating that its immunomodulatory effects are reproducible across distinct models of psoriasiform inflammation. Inflammatory gene expression was subsequently quantified in lesional skin. Compared with controls, IMQ markedly upregulated *Il17a*, *Il17e*, *Il17f*, *Il22*, and the Th17-lineage transcription factor *Rorc*. Nomilin significantly reduced the expression of these genes, consistent with the suppression of pathogenic Th17-associated responses observed by flow cytometry (Fig. 2D). Similar transcriptional changes were observed in the TPA-induced model, in which nomilin likewise suppressed the expression of Th17-associated cytokines and inflammatory mediators in lesional skin (Supplementary Fig. S2F). These findings indicate that nomilin consistently attenuates pathogenic Th17-driven inflammatory programs across distinct models of psoriasiform inflammation.

### Nomilin restrains immunometabolic activation and mTORC1 signaling in psoriasiform skin

Psoriatic inflammation is closely associated with metabolic reprogramming characterized by enhanced glucose utilization and mitochondrial dysfunction 8, 9, 18. Consistent with this concept, IMQ-induced psoriasiform lesions exhibited markedly elevated glucose uptake, as indicated by increased 2-NBDG incorporation compared with control skin (Fig. 3A). Expression of the glucose transporter *Glut1* was likewise significantly increased and was markedly suppressed following nomilin treatment (Fig. 3B). IMQ stimulation also increased mitochondrial mass (MitoTracker^+^) and mitochondrial ROS (MitoTracker^+^MitoSOX^+^) production, as assessed by MitoTracker^+^ and MitoSOX^+^ staining, respectively. Nomilin markedly reduced both mitochondrial accumulation and mitochondrial ROS generation within lesional skin cells (Fig. 3C-D), indicating attenuation of metabolic hyperactivation. Seahorse extracellular flux analysis further revealed substantial bioenergetic alterations in psoriasiform lesions. IMQ stimulation significantly increased oxygen consumption rate (OCR), as reflected by elevated basal respiration, ATP production, and maximal respiration, all of which were attenuated by nomilin treatment (Fig. 3E). In contrast, spare respiratory capacity was not affected. Extracellular acidification rate (ECAR) analysis similarly demonstrated enhanced glycolytic activity in lesional skin. Nomilin markedly reduced non-glycolytic acidification, glycolysis, and glycolytic capacity, whereas glycolytic reserve was unchanged (Fig. 3F). Given the central role of mTOR signaling in metabolic regulation, we next examined mTORC1 pathway activation in lesional skin (Fig. 3G). IMQ stimulation markedly increased phosphorylation of mTOR and its downstream targets RAPTOR, p70S6K, and 4EBP1. Nomilin significantly reduced phosphorylation of all mTORC1 signaling components, demonstrating effective inhibition of mTORC1 activation *in vivo* (Fig. 3H). In parallel, nomilin enhanced AMPK phosphorylation and increased DDIT4 expression (Fig. 3I), suggesting that suppression of metabolic activation is associated with modulation of the DDIT4-AMPK-mTORC1 signaling axis.

Similar metabolic alterations were observed in the TPA-induced model. Nomilin significantly reduced glucose uptake, Glut1 expression, mitochondrial mass, mitochondrial ROS production, oxidative phosphorylation, and glycolytic activity in TPA-induced lesions (Supplementary Fig. S3A-H). Consistent with these findings, nomilin suppressed mTORC1 activation while enhancing AMPK phosphorylation and DDIT4 expression (Supplementary Fig. S3I-L). Molecular docking analysis further predicted that nomilin binds the FKBP12-associated FRB domain of mTOR through interactions involving His87, Arg57, and Glu54 (Supplementary Fig. S3M). Consistent with this prediction, pull-down analysis confirmed a physical interaction between nomilin and mTOR (Supplementary Fig. S3N). Taken together, these findings demonstrate that nomilin suppresses psoriatic immunometabolic activation by attenuating glucose metabolism, mitochondrial dysfunction, and mTORC1 signaling while promoting DDIT4 and AMPK activation.

### Nomilin inhibits IL-17A-induced metabolic activation in primary human keratinocytes

To investigate whether nomilin regulates inflammatory metabolic reprogramming in human psoriasis-relevant cells, we isolated primary human keratinocytes from biopsy skin samples and stimulated them with IL-17A in the presence or absence of nomilin or rapamycin (Fig. 4A). Nomilin up to 50 µg/ml did not impair cell viability, indicating that the concentrations used were well tolerated *in vitro* (Fig. 4B). We first examined the expression of psoriasis-associated genes in IL-17A-stimulated cells by qPCR (Fig. 4C). IL-17A markedly increased the expression of psoriasis-associated genes, including *DEFB4*, *LCN2*, *S100A7*, *S100A8*, and *S100A9*. Nomilin significantly suppressed the induction of these inflammatory mediators and generally exhibited inhibitory effects comparable to or greater than those observed with rapamycin (Fig. 4C). Similar anti-inflammatory effects were observed for pro-inflammatory cytokines, as nomilin significantly reduced IL1β and IL6 expression in IL-17A-stimulated keratinocytes (Supplementary Fig. S4). Consistent with these transcriptional changes, IL-17A stimulation significantly increased glucose uptake, as indicated by elevated 2-NBDG incorporation, whereas nomilin markedly reduced both the frequency and absolute number of 2-NBDG⁺ cells (Fig. 4D-E).

Mitochondrial alterations were next examined in IL-17A-stimulated keratinocytes. IL-17A increased the proportion of dysfunctional mitochondria, characterized by CMXROS^low^MitoTracker^high^ cells, whereas nomilin significantly restored mitochondrial integrity (Fig. 4F). Ultrastructural analysis further revealed the accumulation of morphologically damaged mitochondria in IL-17A-stimulated keratinocytes, characterized by swollen mitochondria and disrupted cristae structures. Nomilin treatment markedly reduced the number of these abnormal mitochondria, indicating preservation of mitochondrial integrity (Fig. 4G). In parallel, IL-17A markedly increased mitochondrial ROS (MitoSOX^+^MitoTracker^+^) production, as assessed by MitoSOX staining, and this effect was significantly attenuated by nomilin (Fig. 4H). These findings indicate that nomilin suppresses IL-17A-induced mitochondrial dysfunction and oxidative stress in primary human keratinocytes. To determine whether these metabolic alterations were associated with changes in mitochondrial respiratory complexes, representative OXPHOS proteins were analyzed by immunoblotting. No substantial changes were observed in CI-NDUFB8, CII-SDHB, or CV-ATP5A among the experimental groups. In contrast, nomilin treatment was associated with relatively increased expression of CIII-UQCRC2 and CIV-MTCO1 compared with IL-17A stimulation alone (Fig. 4I), suggesting selective modulation of mitochondrial respiratory complexes rather than global suppression of mitochondrial proteins.

IL-17A enhanced phosphorylation of mTOR and downstream mTORC1 targets, including RAPTOR, p70S6K, and 4EBP1. Both nomilin and rapamycin markedly suppressed mTORC1 activation, as evidenced by reduced phosphorylation of mTOR, RAPTOR, p70S6K, and 4EBP1 (Fig. 4J). Concomitantly, phosphorylation of AMPK was increased, and DDIT4 expression was upregulated in both treatment groups. These findings are consistent with the *in vivo* observations, indicating that nomilin and rapamycin similarly restrain IL-17A-driven mTORC1 signaling while engaging AMPK-DDIT4 regulatory pathways in primary human keratinocytes.

### DDIT4 is required for Nomilin-mediated suppression of inflammatory signaling

Because nomilin consistently increased DDIT4 expression both *in vivo* and *in vitro*, we next assessed whether DDIT4 mediates the anti-inflammatory and metabolic effects of nomilin in primary human keratinocytes. Cells were transfected with DDIT4-specific siRNA prior to IL-17A stimulation in the presence or absence of nomilin (Fig. 5A). Efficient DDIT4 silencing was validated at both mRNA and protein levels (Supplementary Fig. S5A). In siCtrl-transfected cells, IL-17A robustly induced psoriasis-associated transcripts, including *DEFB4*, *LCN2*, *S100A7*, *S100A8*, and *S100A9*. Nomilin significantly attenuated this transcriptional induction. In contrast, although DDIT4 silencing itself reduced the magnitude of the IL-17A-induced transcriptional response, nomilin no longer produced a significant additional inhibitory effect in DDIT4-silenced cells (Fig. 5A). These findings suggest that DDIT4 is required for the full inhibitory activity of nomilin on IL-17A-induced inflammatory gene expression.

IL-17A stimulation also promoted mitochondrial oxidative stress, reflected by a marked increase in the MitoSOX⁺MitoTracker⁺ population. Nomilin significantly reduced mitochondrial ROS accumulation in siCtrl-transfected cells; however, this protective effect was largely lost following DDIT4 knockdown, resulting in persistently elevated mitochondrial ROS levels despite nomilin treatment (Fig. 5B). Confocal microscopy further corroborated these findings. IL-17A stimulation increased MitoTracker and MitoSOX fluorescence intensities and enhanced mitochondrial ROS accumulation, whereas nomilin markedly reduced both signals in siCtrl-transfected cells. In contrast, DDIT4-silenced cells remained refractory to nomilin treatment and retained elevated mitochondrial ROS and mitochondrial staining patterns comparable to those observed following IL-17A stimulation alone (Fig. 5C). These findings indicate that DDIT4 is required for nomilin-mediated restoration of mitochondrial homeostasis under inflammatory conditions. Consistent with these observations, IL-17A markedly increased phosphorylation of mTOR and its downstream targets RAPTOR and p70S6K. Nomilin effectively suppressed mTORC1 activation in siCtrl-transfected cells, whereas this inhibitory effect was abolished following DDIT4 silencing, resulting in sustained phosphorylation of mTORC1 signaling components despite nomilin treatment (Fig. 5D). Although nomilin enhanced AMPK phosphorylation, its anti-inflammatory effects were largely preserved following AMPK knockdown (Supplementary Fig. S5B), indicating that AMPK is dispensable for the anti-inflammatory actions of nomilin. Collectively, these findings demonstrate that DDIT4, rather than AMPK, is required for nomilin-mediated suppression of inflammatory gene expression, mitochondrial oxidative stress, and mTORC1 signaling in IL-17A-stimulated primary human keratinocytes.

### DDIT4-dependent suppression of mTOR signaling underlies the anti-psoriatic effects of nomilin *in vivo*

Molecular docking analysis predicted that nomilin binds DDIT4 with a binding affinity of -9.0 kcal/mol and an estimated Ki of 252.91 nM, involving hydrogen-bond interactions with Gln153, Ser111, and Leu109, together with hydrophobic interactions involving Trp186 and Ile189 (Fig. 6A). To experimentally evaluate target engagement, we performed a cellular thermal shift assay (CETSA), which assesses compound-protein interactions based on ligand-induced thermal stabilization of the target protein. CETSA demonstrated that nomilin markedly increased the thermal stability of DDIT4, resulting in enhanced preservation of DDIT4 protein at elevated temperatures compared with vehicle-treated controls (Fig. 6B). These findings provide biochemical evidence supporting direct interaction between nomilin and DDIT4 under cellular conditions.

IMQ-induced psoriasiform inflammation was established in mice receiving intradermal siCtrl or siDDIT4 administration together with nomilin treatment (Fig. 6C). Efficient knockdown of DDIT4 was confirmed by immunoblot analysis of skin tissue (Fig. 6D). Nomilin markedly improved erythema, scaling, and tissue thickening in siCtrl-injected mice, whereas these therapeutic effects were largely abolished following DDIT4 silencing (Fig. 6E). Consistent with these observations, nomilin significantly reduced ear thickness, epidermal thickness, and dermal thickness in siCtrl- injected mice, whereas these improvements were lost in siDDIT4-injected mice. Inflammatory gene expression exhibited a similar pattern. Nomilin significantly suppressed the expression of *Tnfα*, *Il1β*, *S100a7*, *Defb4*, *Rorc*, and *Il17a* in siCtrl-injected mice. In contrast, DDIT4 knockdown largely abolished these inhibitory effects, resulting in persistently elevated inflammatory gene expression despite nomilin treatment (Fig. 6F). mTOR signaling was likewise dependent on DDIT4. Nomilin markedly reduced phosphorylation of mTOR and p70S6K in IMQ-induced lesions, whereas DDIT4 silencing prevented suppression of both signaling molecules despite nomilin treatment (Fig. 6G). Together, these findings identify DDIT4 as a critical mediator of nomilin activity and demonstrate that DDIT4-dependent inhibition of mTOR signaling is required for the anti-inflammatory effects of nomilin in psoriasiform skin inflammation.

### Nomilin inhibits pathogenic Th17 responses through DDIT4-dependent mTOR suppression

The DDIT4-mTOR axis identified in keratinocytes and psoriasiform lesions is also a central regulator of pathogenic Th17 responses. Nomilin treatment did not affect the viability of primary CD4⁺ T cells at the concentrations used for subsequent experiments (Supplementary Fig. S6A). Naïve CD4⁺ T cells were therefore differentiated under Th17-polarizing conditions in the presence or absence of nomilin (Fig. 7A). Th17-polarizing conditions efficiently induced the generation of IL-17A^+^-producing cells, whereas nomilin markedly reduced Th17 differentiation (Fig. 7B). In line with this observation, phosphorylation of mTOR and its downstream target p70S6K was significantly increased during Th17 differentiation and was markedly suppressed by nomilin treatment (Fig. 7C and Supplementary Fig. S6B), indicating that nomilin directly restrains mTOR-dependent Th17 programming.

The immunomodulatory effects of nomilin were next evaluated in PBMCs obtained from healthy donors and patients with psoriasis (Fig. 7D). Nomilin did not affect PBMC viability at the concentration used for subsequent experiments (Supplementary Fig. S6C). Anti-CD3 stimulation increased the frequencies of TNF-α⁺, IFN-γ⁺, and IL-17A⁺ CD4⁺ T cells in both cohorts. Nomilin significantly reduced TNF-α⁺ and IFN-γ⁺ CD4⁺ T cells in healthy donors and psoriasis patients (Fig. 7E). Nomilin also reduced the frequency of IL-17A⁺ CD4⁺ T cells, with statistical significance observed in psoriasis-derived PBMCs but not in healthy donor samples. Consistent with these cellular effects, anti-CD3 stimulation induced robust activation of mTORC1 signaling in PBMCs, as evidenced by increased phosphorylation of mTOR, RAPTOR, and p70S6K. Nomilin markedly suppressed phosphorylation of all three signaling components (Fig. 7F and H). In parallel, nomilin significantly increased DDIT4 expression in activated PBMCs (Fig. 7G-H), further supporting engagement of the DDIT4-mTOR signaling axis. Together, these findings demonstrate that nomilin suppresses pathogenic T-cell activation and Th17 differentiation through inhibition of mTOR signaling and induction of DDIT4, extending its immunometabolic regulatory effects from keratinocytes and experimental models to human psoriasis-associated immune responses.

## Discussion

This study demonstrates that nomilin does not merely suppress psoriasiform inflammation, but rather restores immunometabolic homeostasis through DDIT4-dependent inhibition of mTORC1 signaling. These findings align with the emerging paradigm that psoriasis is not solely a cytokine-driven disorder, but a disease sustained by persistent immunometabolic hyperactivation 18. Although two doses of nomilin were evaluated in this study, their overall therapeutic effects were largely comparable, and no clear dose-dependent differences were observed across the clinical, histological, and molecular parameters examined.

In support of this concept, consistent increases in glucose uptake, glycolytic activity, oxidative metabolism, and mitochondrial ROS production were observed in both TPA- and IMQ-induced psoriasiform lesions. These metabolic alterations are increasingly recognized not simply as consequences of inflammation but as active drivers that sustain inflammatory responses 18, 43. The metabolic alterations observed here are consistent with previous reports 43, and nomilin broadly reduced glucose utilization, mitochondrial accumulation, oxidative phosphorylation, glycolysis, and mitochondrial ROS production. Notably, nomilin attenuated both glycolytic and mitochondrial bioenergetic programs simultaneously, suggesting restoration of pathological immunometabolic activation rather than selective inhibition of an individual metabolic pathway. Under inflammatory conditions, immune cells and keratinocytes are known to exhibit a hypermetabolic state characterized by simultaneous increases in glycolytic activity and mitochondrial respiration. Therefore, the concomitant reductions in glycolysis and OXPHOS observed following nomilin treatment are more consistent with normalization of inflammation-associated metabolic hyperactivation than with nonspecific metabolic suppression.

mTOR signaling functions as a central regulatory hub integrating nutrient sensing, cellular metabolism, and inflammatory responses 43. Nomilin consistently inhibited phosphorylation of mTOR and its downstream effectors RAPTOR, p70S6K, and 4EBP1 in psoriasiform lesions, primary human keratinocytes, Th17 cells, and activated PBMCs. Concomitantly, nomilin induced expression of DDIT4, a stress-responsive negative regulator of mTORC1 that is activated under conditions of metabolic and oxidative stress 44, 45. Importantly, both *in vitro* and *in vivo* loss-of-function studies established DDIT4 as an essential mediator of nomilin activity. Silencing DDIT4 abolished the anti-inflammatory, metabolic, and mTOR-suppressive effects of nomilin in primary keratinocytes and IMQ-induced psoriasiform lesions, demonstrating that DDIT4 is functionally indispensable for this response. Furthermore, molecular docking predicted a favorable interaction between nomilin and DDIT4, whereas CETSA analysis supported target engagement under cellular conditions. Collectively, these findings position DDIT4 as a critical molecular node linking nomilin treatment to suppression of mTOR-driven immunometabolic activation.

A major conceptual advance of the present study is the identification of a DDIT4-centered immunometabolic regulatory axis underlying the therapeutic effects of nomilin. Although modulation of mTOR signaling and metabolic activation has been reported for several natural compounds, including limonoids, the upstream regulatory mechanisms responsible for these effects have remained poorly defined 46, 47. Notably, our previous study using limonin demonstrated inhibition of mTOR signaling and metabolic activity but did not identify induction of DDIT4, nor did it include genetic loss-of-function validation 46. In contrast, the present study demonstrates that DDIT4 is consistently induced by nomilin across psoriasiform lesions, primary keratinocytes, Th17 cells, and activated PBMCs. Moreover, genetic silencing of DDIT4 abolished nomilin-mediated suppression of mTOR signaling, mitochondrial ROS accumulation, inflammatory gene expression, and psoriasiform inflammation in both *in vitro* and *in vivo* settings. Thus, the principal advance of this study lies not in the observation of mTOR inhibition itself, but in establishing the DDIT4-mTOR axis as an essential regulatory pathway governing nomilin-mediated immunometabolic reprogramming. These findings provide a mechanistic framework linking nomilin to immunometabolic regulation in psoriasis.

Interestingly, nomilin also increased AMPK phosphorylation. However, suppression of inflammatory gene expression was largely preserved following AMPK silencing, whereas DDIT4 silencing completely abrogated the biological activity of nomilin. Previous studies have shown that DDIT4 can regulate mTORC1 through mechanisms that are at least partially independent of AMPK 48, 49. Accordingly, our findings suggest that DDIT4-dependent regulation represents the dominant pathway mediating the anti-inflammatory effects of nomilin, whereas AMPK activation may serve as a complementary regulatory mechanism.

Mitochondrial ROS act as signaling amplifiers, enhancing NF-κB and mTOR pathways and contributing to the persistence of inflammation 50, 51. Nomilin significantly reduced mitochondrial ROS accumulation in both lesional tissue and IL-17A-stimulated primary human keratinocytes while simultaneously restoring mitochondrial integrity. Notably, nomilin partially restored the expression of specific mitochondrial respiratory chain components, including UQCRC2 and MTCO1. However, the abundance of respiratory chain complex proteins does not necessarily correlate directly with functional respiratory activity, suggesting that these changes may reflect improved mitochondrial fitness and quality control rather than enhanced OXPHOS. Consistent with previous reports showing that inflammatory stimulation can increase MitoTracker Green fluorescence, which has been interpreted as reflecting the accumulation of dysfunctional mitochondria rather than expansion of healthy mitochondrial mass 52, our findings further support the interpretation that nomilin alleviates mitochondrial dysfunction under inflammatory conditions. These observations suggest that suppression of mitochondrial dysfunction and inhibition of mTOR signaling are interconnected outcomes within a coordinated immunometabolic regulatory network rather than independent biological events.

At the immune cell level, nomilin reduced neutrophil and macrophage accumulation in lesional skin and draining lymph nodes while suppressing Th1 and Th17 responses and expanding Foxp3⁺ regulatory T cell populations. Importantly, nomilin directly inhibited Th17 differentiation and attenuated mTOR signaling during Th17 polarization, indicating that its effects extend beyond keratinocytes to pathogenic adaptive immune programming. In PBMCs from healthy donors and patients with psoriasis, nomilin broadly reduced TNF-α and IFN-γ production. Although suppression of IL-17A-producing CD4⁺ T cells reached statistical significance only in psoriasis-derived PBMCs, nomilin reduced activated T-cell responses in both cohorts. Because baseline Th17-associated responses were elevated in psoriasis patients, definitive conclusions regarding disease-selective responsiveness cannot be drawn from the present data. Collectively, nomilin restrains both innate and adaptive immune circuits that sustain chronic psoriatic inflammation. These observations are highly relevant to current concepts of psoriasis pathogenesis. Psoriasis is sustained by reciprocal interactions between activated keratinocytes and IL-23/Th17-driven immune responses, resulting in persistent production of inflammatory cytokines, epidermal hyperplasia, and chronic tissue remodeling 53, 54. Increasing evidence indicates that metabolic reprogramming and oxidative stress amplify these pathogenic circuits 55. Therefore, the ability of nomilin to simultaneously regulate keratinocyte activation, Th17-associated responses, mitochondrial dysfunction, and mTOR-dependent metabolic signaling suggests that its therapeutic effects target multiple interconnected drivers of psoriasis pathogenesis rather than a single inflammatory pathway.

Rapamycin also effectively reduced mTORC1 phosphorylation and suppressed inflammatory markers in this study, further reinforcing the central role of mTORC1 in sustaining psoriatic inflammation 18. However, broad mTOR inhibition can globally restrict immune cell proliferation and differentiation, potentially disrupting immune homeostasis during prolonged treatment 18. Conversely, nomilin, acting through a DDIT4-dependent mechanism, modulated immunometabolic activation associated with inflammatory responses. This distinction highlights its potential as a disease-context-specific metabolic regulator rather than a broad immunosuppressive agent.

Several limitations of this study should be acknowledged. Although both TPA- and IMQ-induced mouse models reproduce key features of psoriasiform inflammation, they do not fully recapitulate the complexity and chronicity of human psoriasis. In addition, while patient-derived PBMCs were included to enhance translational relevance, the clinical cohort remained relatively limited. Therefore, larger clinical studies will be necessary to further evaluate the therapeutic potential of nomilin and determine whether modulation of the DDIT4-mTOR axis can be translated into an effective therapeutic strategy for psoriasis. Furthermore, although nomilin was administered intraperitoneally in the present study, topical delivery may represent a clinically attractive approach for psoriasis, warranting future investigation of formulation optimization, skin penetration, and local therapeutic efficacy.

## Conclusion

This study demonstrates that nomilin, a citrus-derived tetranortriterpenoid limonoid, alleviates psoriasis-like inflammation by correcting immunometabolic hyperactivation through DDIT4-dependent suppression of mTORC1 signaling and reduction of mitochondrial ROS. By reducing glucose utilization, mitochondrial dysfunction, and mTORC1 activity while simultaneously restraining pathogenic Th17 responses, nomilin reprograms the metabolic state that sustains chronic inflammation rather than merely suppressing downstream inflammatory cytokines. Importantly, genetic loss-of-function studies established DDIT4 as an essential upstream regulator of nomilin activity and identified the DDIT4-mTOR axis as a key functional node governing psoriasiform inflammation. These findings extend beyond descriptive observations of mTOR inhibition and provide a mechanistic framework linking nomilin to immunometabolic regulation in psoriasis. Collectively, our results position nomilin as a promising immunometabolism-targeted therapeutic candidate and highlight the DDIT4-mTOR axis as a potential therapeutic target for psoriasis.

## Supplementary Material

Supplementary materials and methods, figures and tables.

## Figures and Tables

**Figure 1:**
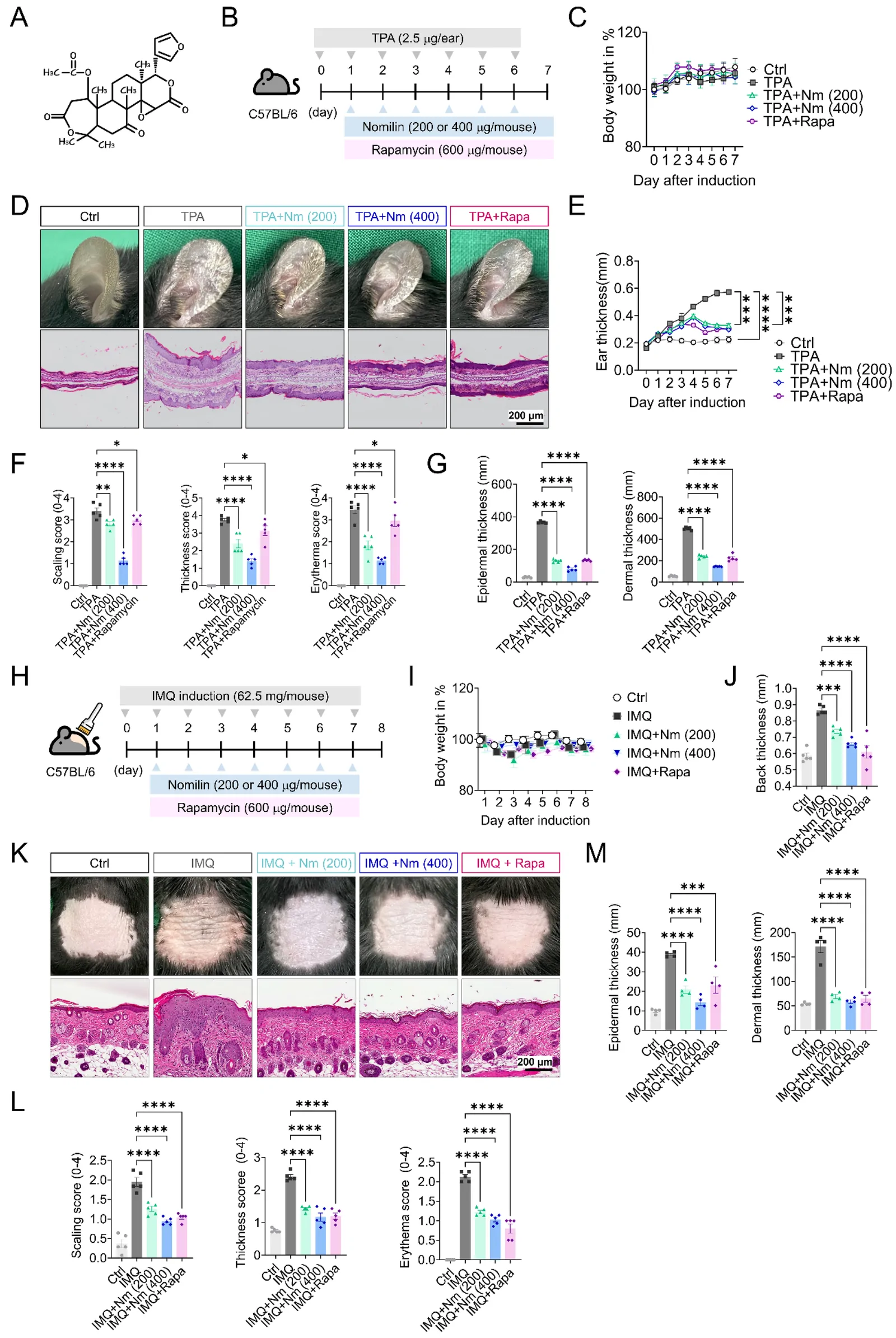
** Nomilin alleviates psoriasiform skin inflammation in a TPA- and IMQ-induced mouse models.** C57BL/6 mice were subjected to either a TPA-induced acute psoriasiform dermatitis model or an IMQ-induced psoriasis-like skin inflammation model and treated with nomilin (Nm; 200 or 400 μg per mouse, intraperitoneally) or rapamycin (600 μg per mouse).** (A)** Chemical structure of nomilin. **(B-G)** TPA-induced psoriasiform dermatitis model. Mice received topical application of 12-O-tetradecanoylphorbol-13-acetate (TPA; 2.5 μg per ear) for 7 consecutive days. Nomilin or rapamycin was administered daily beginning 1 day after TPA induction.** (B)** Experimental scheme. **(C)** Body weight changes during the experimental period are expressed as a percentage of the initial body weight. **(D)** Representative macroscopic images of ear tissues (top panel) and corresponding hematoxylin and eosin (H&E)-stained sections (bottom panel) on day 7. Scale bar, 200 μm. **(E)** Ear thickness is measured daily using a dial thickness gauge. **(F)** Psoriasis Area and Severity Index (PASI) scores during the treatment period. **(G)** Quantification of epidermal and dermal thickness from H&E-stained sections. **(H-L)** IMQ-induced psoriasis-like skin inflammation model. Mice received daily topical application of imiquimod (IMQ; 62.5 mg per mouse) for 8 consecutive days. Nomilin or rapamycin was administered daily beginning 1 day after IMQ induction.** (H)** Experimental scheme. (I) Body weight changes during the experimental period expressed as a percentage of initial body weight. (J) Back skin thickness measured throughout the experiment. (K) Representative macroscopic images of dorsal skin lesions (upper panels) and corresponding H&E-stained sections (lower panels) collected on day 8. Scale bar, 200 μm. (L) Clinical severity scores for scaling, thickness, and erythema. (M) Quantification of epidermal and dermal thickness from H&E-stained sections. Data are presented as mean ± SEM (*n* = 5 mice per group). Statistical significance was determined by one-way ANOVA followed by Holm-Šídák multiple-comparison test. ****P < 0.0001, ***P < 0.001, **P < 0.01, *P < 0.05.

**Figure 2:**
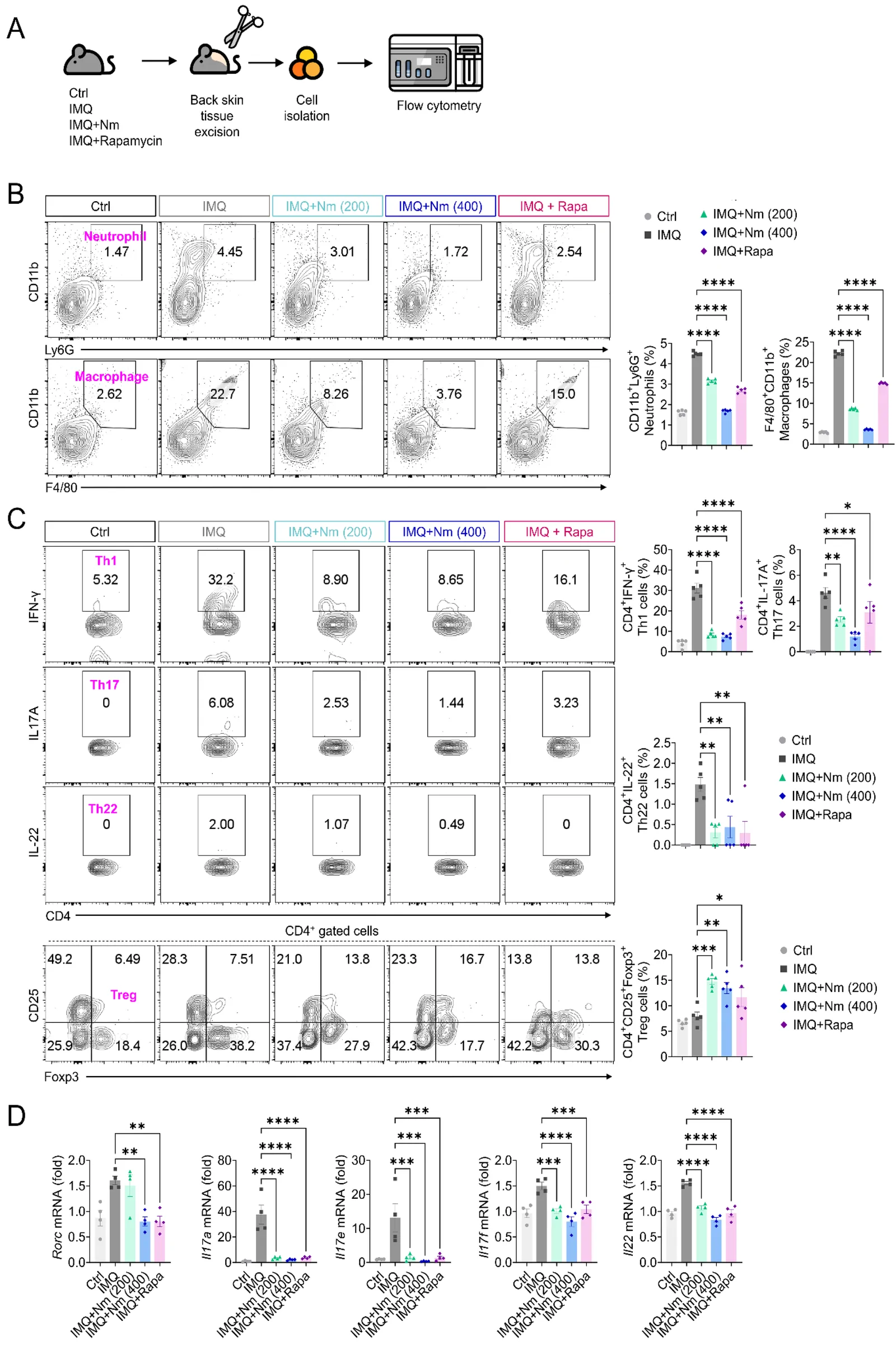
** Nomilin suppresses myeloid and T-cell inflammatory responses in IMQ-induced psoriasiform lesions. (A)** Experimental scheme for immune-cell profiling of IMQ-induced psoriasiform skin lesions. Back skin tissues were harvested, dissociated into single-cell suspensions, and analyzed by flow cytometry. **(B)** Representative flow cytometry plots and quantification of CD11b⁺Ly6G⁺ neutrophils and CD11b⁺F4/80⁺ macrophages in skin lesions. **(C)** Representative flow cytometry plots and quantification of CD4⁺ T-cell subsets, including Th1 (CD4⁺IFN-γ⁺), Th17 (CD4⁺IL-17A⁺), Th22 (CD4⁺IL-22⁺), and regulatory T cells (CD4⁺CD25⁺Foxp3⁺) in lesional skin. **(D)** Relative mRNA expression levels of *Rorc*, *Il17a*, *Il17e*, *Il17f*, and *Il22* in skin lesions, determined by quantitative PCR and expressed relative to the control group. Data are presented as mean ± SEM (*n* = 5 mice per group). Statistical significance was determined by one-way ANOVA followed by Holm-Šídák multiple-comparison test. ****P < 0.0001, ***P < 0.001, **P < 0.01, *P < 0.05.

**Figure 3:**
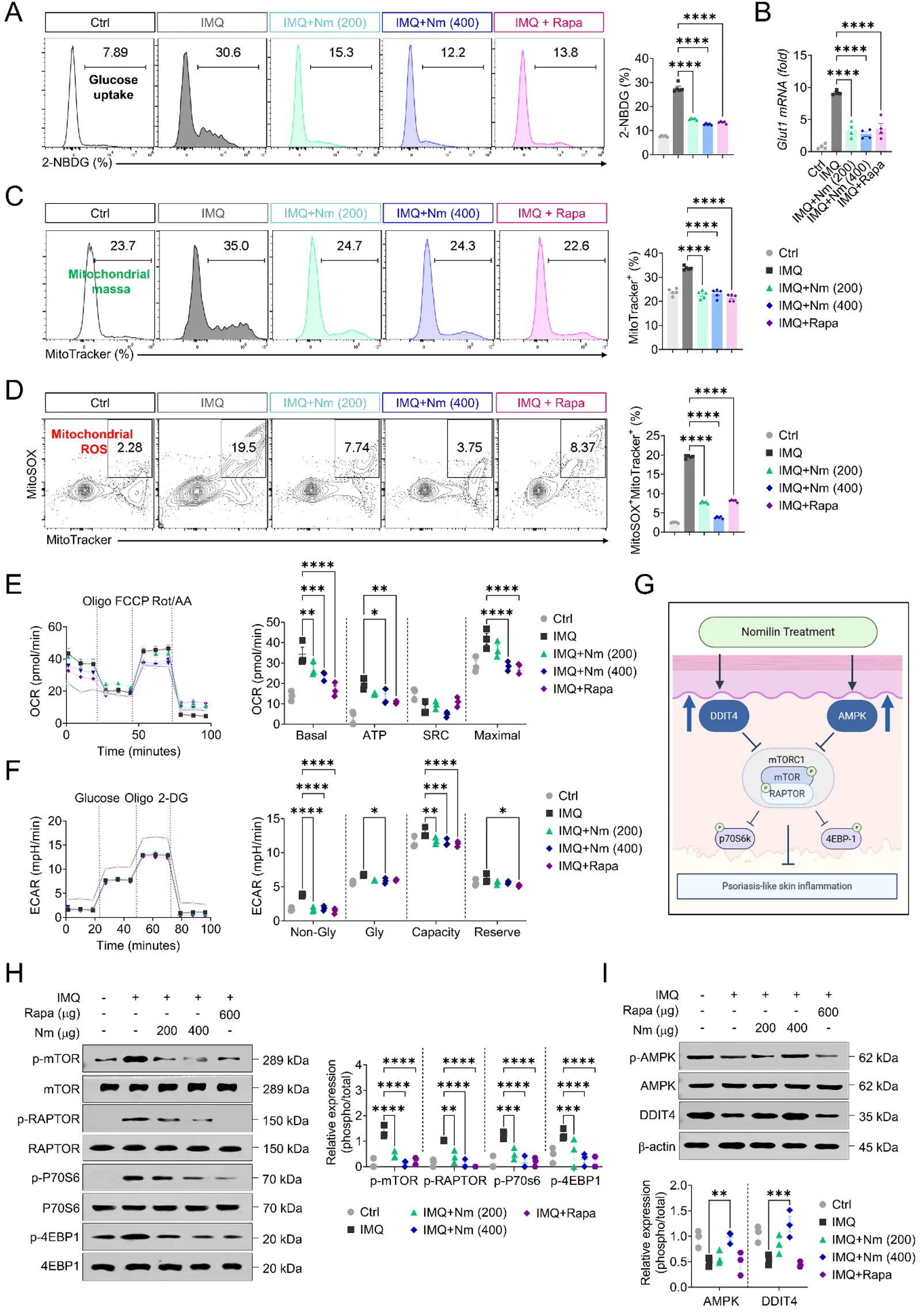
** Nomilin suppresses immunometabolic activation and mTORC1 signaling in IMQ-induced psoriasiform lesions.** C57BL/6 mice were subjected to IMQ-induced psoriasiform skin inflammation and treated with nomilin (Nm; 200 or 400 μg per mouse) or rapamycin (600 μg per mouse) as described in Fig. 1H. **(A)** Representative histograms and quantification of glucose uptake measured by 2-NBDG incorporation in cells isolated from skin lesions. **(B)** Relative *Glut1* mRNA expression in skin lesions determined by quantitative PCR. **(C)** Representative histograms and quantification of mitochondrial mass measured by MitoTracker staining. **(D)** Representative flow cytometry plots and quantification of mitochondrial ROS production measured by MitoTracker and MitoSOX staining. **(E)** Oxygen consumption rate (OCR) profiles of cells isolated from skin lesions (left) and quantification of OCR parameters, including basal respiration, ATP production, spare respiratory capacity (SRC), and maximal respiration (right). **(F)** Extracellular acidification rate (ECAR) profiles (left) and quantification of glycolytic parameters, including non-glycolytic acidification, glycolysis, glycolytic capacity, and glycolytic reserve (right). **(G)** Schematic model illustrating the proposed mechanism by which nomilin suppresses psoriasiform inflammation through activation of DDIT4 and AMPK and subsequent inhibition of mTORC1 signaling. **(H)** Representative Western blotting images and corresponding densitometric quantification of mTORC1 signaling pathway proteins, including phosphorylated mTOR, RAPTOR, p70S6K, and 4EBP1, in skin lesions. **(I)** Representative Western blotting images and corresponding densitometric quantification of phosphorylated AMPK and DDIT4 expression in skin lesions. Data are presented as mean ± SEM (*n* = 5 mice per group). Statistical significance was determined by one-way ANOVA followed by Holm-Šídák multiple-comparison test. ****P < 0.0001, ***P < 0.001, **P < 0.01, *P < 0.05.

**Figure 4:**
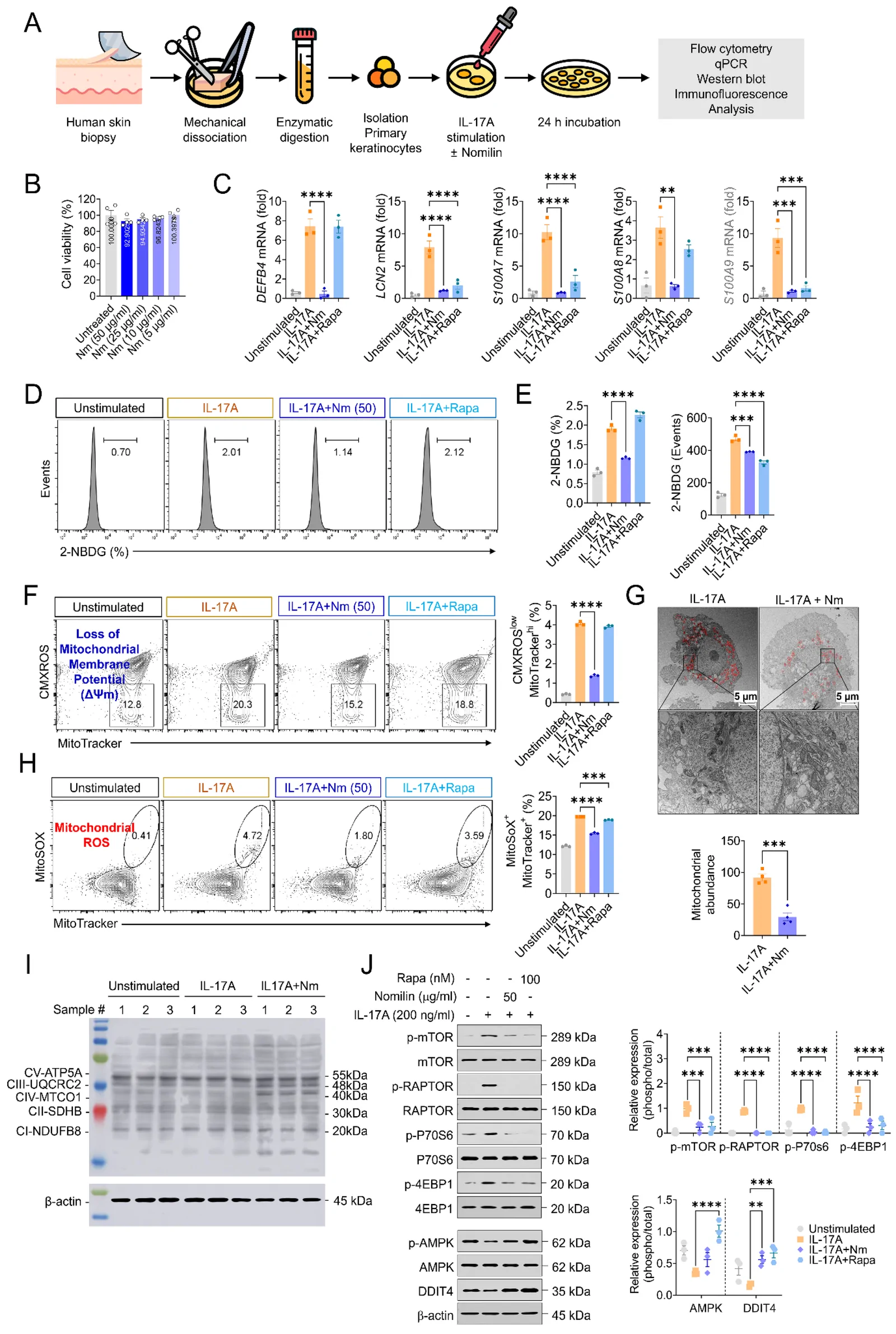
** Nomilin attenuates IL-17A-induced metabolic activation and mTORC1 signaling in primary human keratinocytes.** Primary human keratinocytes were stimulated with IL-17A in the presence or absence of nomilin or rapamycin. **(A)** Experimental scheme illustrating isolation of primary human keratinocytes, IL-17A stimulation, and nomilin treatment. **(B)** Cell viability following nomilin treatment at the indicated concentrations. **(C)** mRNA expression of psoriasis-associated genes (*DEFB4*, *LCN2*, *S100A7*, *S100A8*, and *S100A9*) was analyzed by qPCR and expressed as fold change relative to unstimulated control. **(D)** Representative histograms of 2-NBDG uptake in keratinocytes. **(E)** Bar graphs showing quantification of 2-NBDG⁺ cell frequency and absolute cell number. **(F)** Representative flow cytometry plots and bar graphs showing quantification of mitochondrial integrity assessed by MitoTracker and CMXROS staining. **(G)** Representative transmission electron microscopy (TEM) images showing mitochondrial ultrastructure in IL-17A-stimulated keratinocytes with or without nomilin treatment. Quantification of mitochondrial number per field is shown in the lower panel. Scale bars, 5 μm.** (H)** Representative histograms and bar graphs showing quantification of mitochondrial ROS measured by MitoTracker and MitoSOX staining. **(I)** Representative Western blotting images of mitochondrial electron transport chain subunits (CI-NDUFB8, CII-SDHB, CIII-UQCRC2, CIV-MTCO1, and CV-ATP5A). **(J)** Representative Western blot analysis and corresponding densitometric quantification of phosphorylated mTOR, RAPTOR, p70S6K, and 4EBP1, together with phosphorylated AMPK and DDIT4 expression. Data are presented as mean ± SEM from independent experiments. Statistical significance was determined by one-way ANOVA followed by Holm-Šídák multiple comparison test. ****P < 0.0001, ***P < 0.001, **P < 0.01.

**Figure 5:**
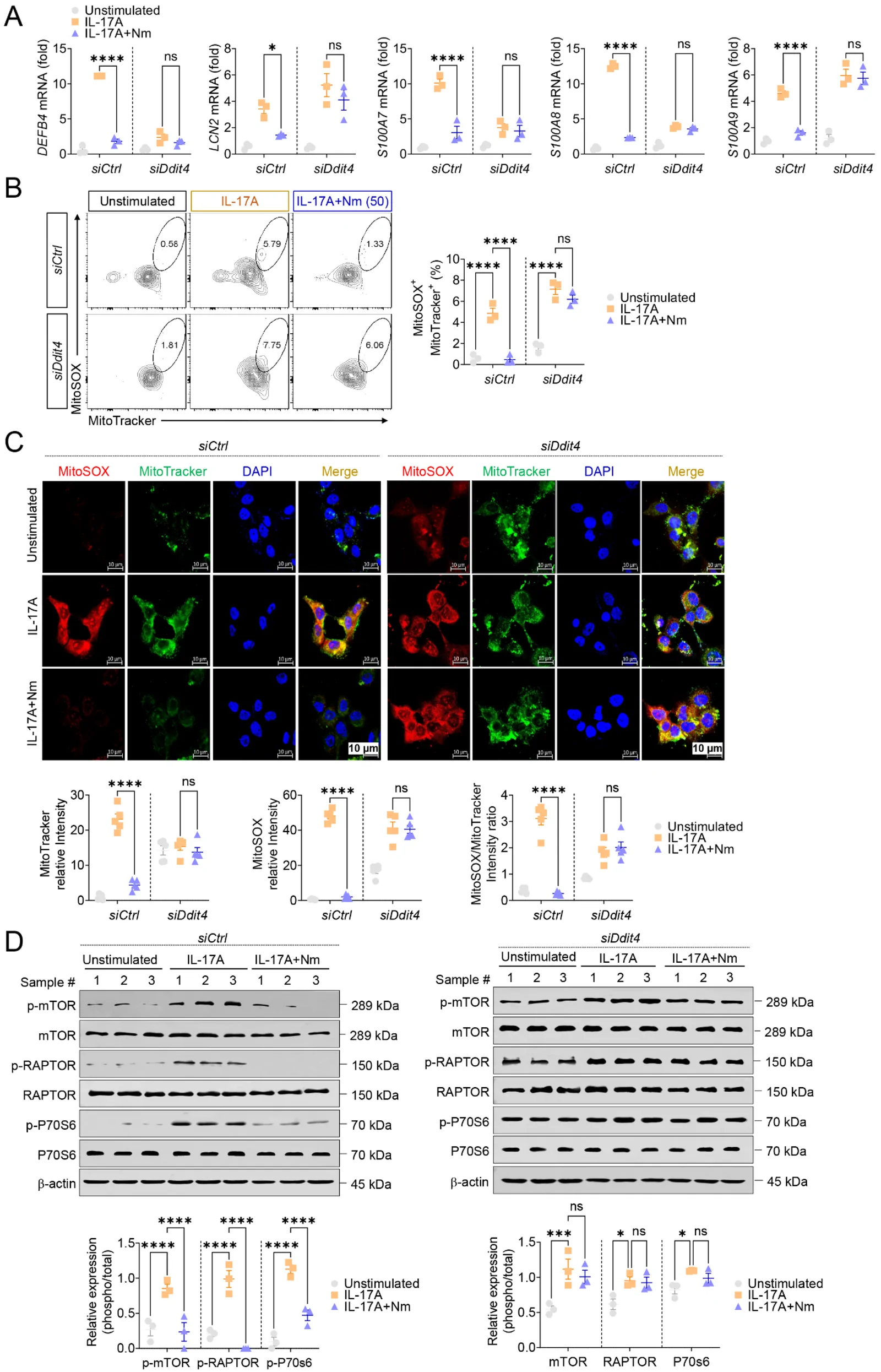
** DDIT4 mediates nomilin-dependent suppression of inflammatory and mTORC1 signaling in primary human keratinocytes. (A-D)** Primary human keratinocytes were transfected with siRNA targeting DDIT4 or control siRNA prior to IL-17A stimulation in the presence or absence of nomilin.** (A)** mRNA expression of psoriasis-associated genes (*DEFB4*, *LCN2*, *S100A7*, *S100A8*, and *S100A9*) was analyzed by qPCR and expressed as fold change relative to unstimulated control.** (B)** Representative flow cytometry plots of mitochondrial ROS assessed by MitoSOX and MitoTracker staining. Bar graphs quantification of MitoSOX⁺MitoTracker⁺ cell populations. **(C)** Representative confocal microscopy images showing mitochondrial ROS (MitoSOX, red), mitochondrial mass (MitoTracker, green), and nuclei (DAPI, blue). Merged images are shown in the right (top) panels. Scale bar, 20 μm. Quantification of MitoSOX intensity, MitoTracker intensity, and MitoSOX⁺MitoTracker⁺ double-positive signals from confocal images (bottom panel). **(D)** Representative Western blotting images showing phosphorylation of mTOR, Raptor, and p70S6K (top). Densitometric quantification of mTOR pathway proteins is shown in (D), normalized to total protein levels (bottom). Data are presented as mean ± SEM from independent experiments. Statistical significance was determined by one-way ANOVA followed by Holm-Šídák multiple comparison test. ****P < 0.0001, ***P < 0.001, *P < 0.05.

**Figure 6:**
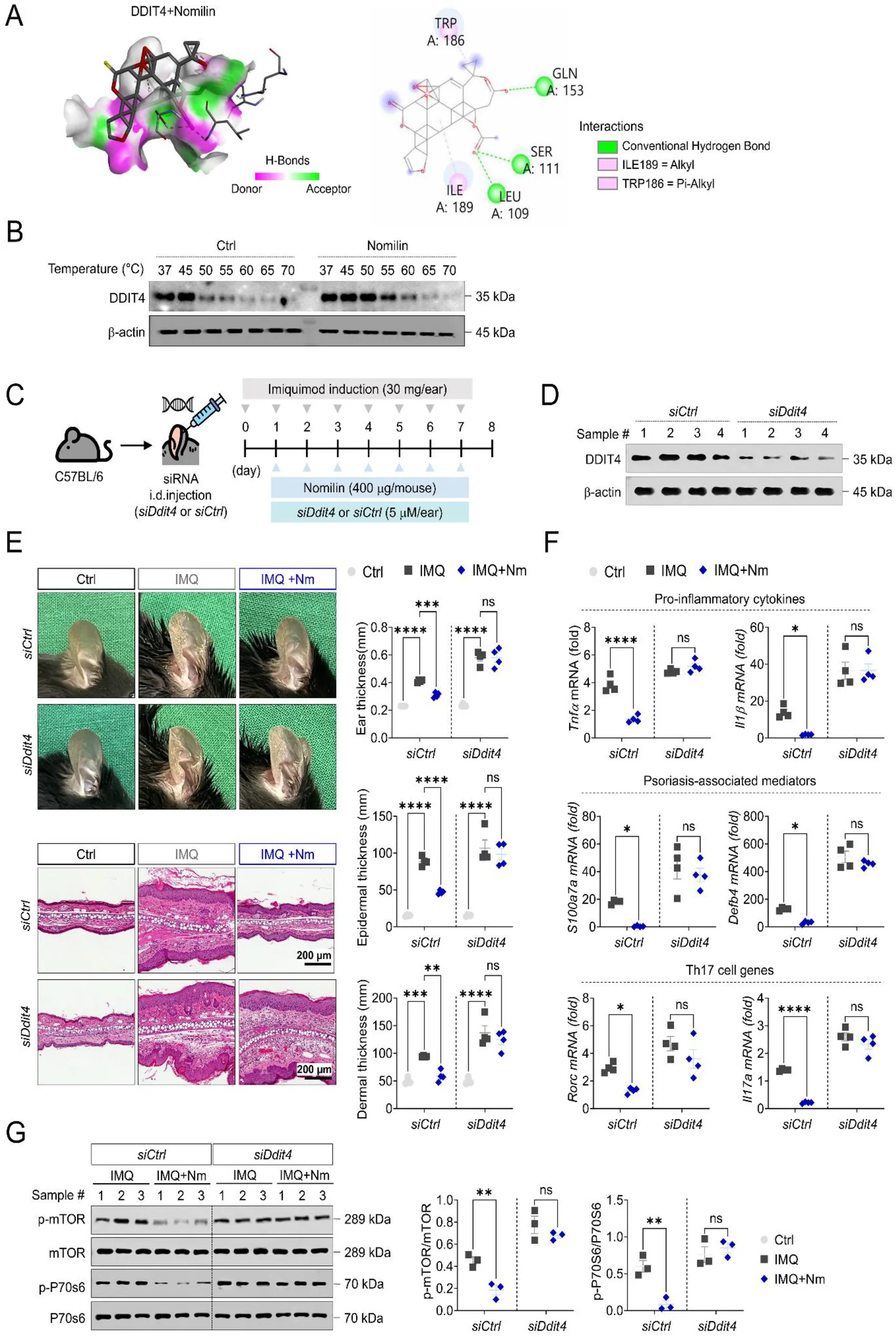
** DDIT4 is required for nomilin-mediated suppression of psoriasiform inflammation *in vivo*. (A)** Molecular docking model predicting the interaction between nomilin and DDIT4. Predicted hydrogen-bonding and hydrophobic interactions are shown. **(B)** Cellular thermal shift assay (CETSA) demonstrating target engagement between nomilin and DDIT4. Primary human keratinocyte lysates were incubated with nomilin and subjected to thermal denaturation. Representative Western blots showing increased thermal stability of DDIT4 in response to nomilin treatment are shown. (C) Experimental scheme for DDIT4 knockdown in the IMQ-induced psoriasis-like skin inflammation model. Mice received intradermal administration of Accell^TM^ siDDIT4 or siCtrl together with nomilin treatment throughout the experimental period. **(D)** Validation of DDIT4 knockdown efficiency in skin lesions by Western blot analysis. **(E)** Representative macroscopic images and H&E-stained sections of skin lesions from siCtrl- and siDDIT4-treated mice. Quantification of ear thickness, epidermal thickness, and dermal thickness is shown in the right panels. Scale bars, 200 μm. **(F)** Relative mRNA expression levels of pro-inflammatory cytokines (*Tnfα* and *Il1β*), psoriasis-associated mediators (*S100a7a* and *Defb4*), and Th17-related genes (*Rorc* and *Il17a*) in skin lesions. **(G)** Representative Western blot analysis and corresponding densitometric quantification of phosphorylated mTOR and p70S6K in skin lesions following DDIT4 silencing. Data are presented as mean ± SEM (*n* = 4 mice per group). Statistical significance was determined by one-way ANOVA followed by Holm-Šídák multiple-comparison test. ****P < 0.0001, ***P < 0.001, **P < 0.01, *P < 0.05.

**Figure 7:**
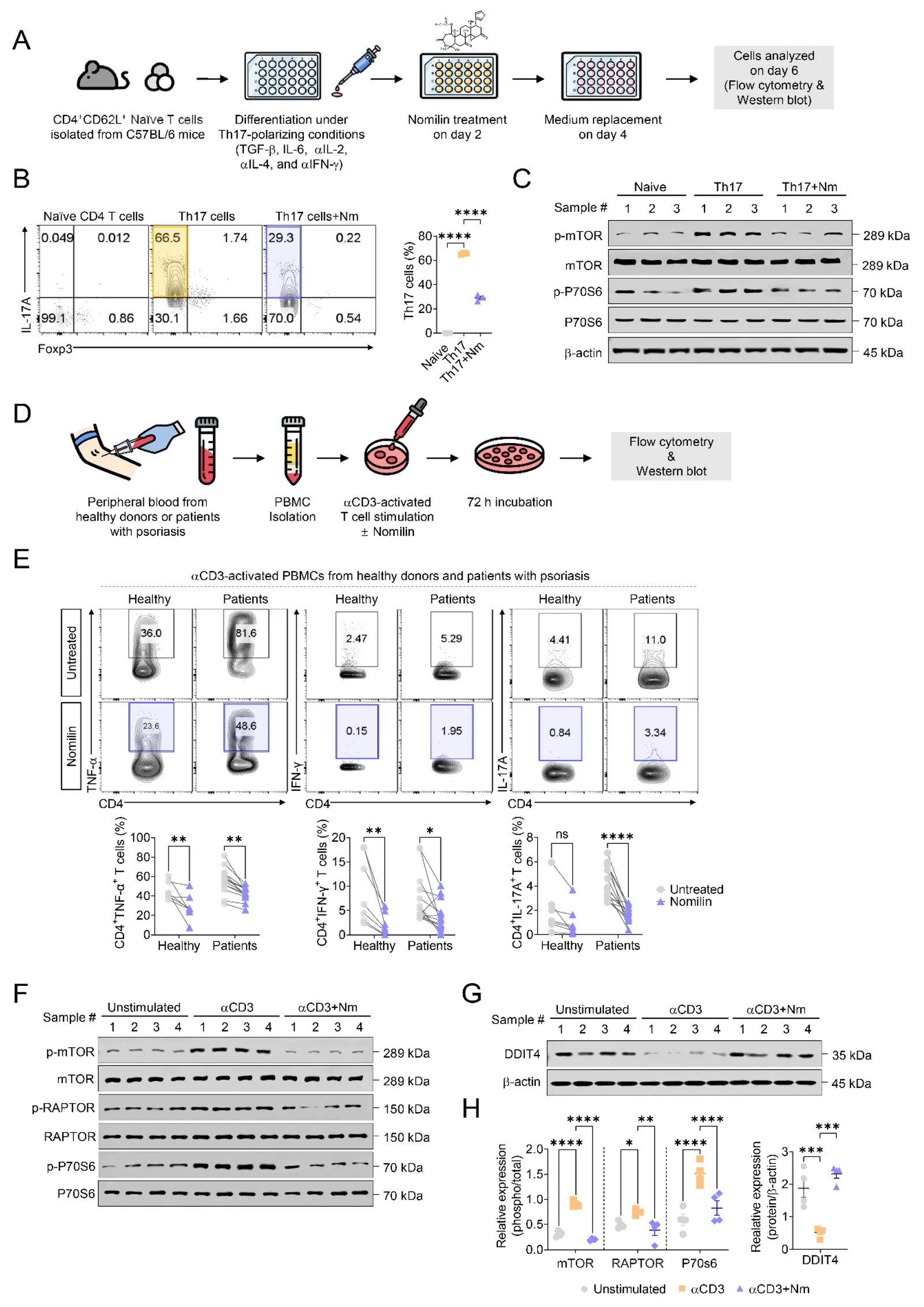
** Nomilin suppresses Th17 differentiation and pathogenic T-cell responses through inhibition of mTOR signaling. (A)** Experimental scheme for *in vitro* Th17 differentiation. Naïve CD4⁺CD62L⁺ T cells isolated from C57BL/6 mice were cultured under Th17-polarizing conditions and treated with nomilin. Cells were analyzed on day 6 by flow cytometry and Western blot analysis.** (B)** Representative flow cytometry plots and quantification of IL-17A-producing Th17 cells differentiated in the presence or absence of nomilin.** (C)** Representative Western blot analysis of mTOR signaling pathway proteins, including phosphorylated mTOR and p70S6K, in naïve CD4⁺ T cells, differentiated Th17 cells, and nomilin-treated Th17 cells. **(D)** Experimental scheme for PBMC isolation, anti-CD3 stimulation, and nomilin treatment. PBMCs from healthy donors (*n* = 7) and patients with psoriasis (*n* = 21) were stimulated with anti-CD3 (1 μg/ml) in the presence or absence of nomilin and subsequently analyzed by flow cytometry (Healthy *n* = 7 vs Patients *n* = 14) and Western blotting (Patients *n* = 7). **(E)** Representative flow cytometry plots and quantification of TNF-α⁺, IFN-γ⁺, and IL-17A⁺ CD4⁺ T cells from healthy donors and patients with psoriasis following anti-CD3 stimulation with or without nomilin treatment. **(F)** Representative Western blot analysis of mTOR signaling pathway proteins, including phosphorylated mTOR, RAPTOR, and p70S6K, in activated human PBMCs. **(G)** Representative Western blot analysis of DDIT4 expression in activated human PBMCs. **(H)** Corresponding densitometric quantification of phosphorylated mTOR, RAPTOR, p70S6K, and DDIT4 expression shown in (F) and (G). Data are presented as mean ± SEM from independent experiments or individual donors. Statistical significance was determined by one-way ANOVA, two-way ANOVA, or paired t-test with appropriate multiple-comparison correction. ****P < 0.0001, ***P < 0.001, **P < 0.01, *P < 0.05.

## Data Availability

Data will be made available on request.

## Abbreviations

2-DG: 2-Deoxy-D-glucose
2-NBDG: 2-[N-(7-nitrobenz-2-oxa-1,3-diazol-4-yl) amino]-2-deoxy-D-glucose
4E-BP1: Eukaryotic translation initiation factor 4E-binding protein 1
ACK: Ammonium-chloride-potassium
AMPK: AMP-activated protein kinase
ATP: Adenosine triphosphate
BPE: Bovine pituitary extract
CIII-UQCRC2: Cytochrome c reductase core protein 2 (Complex III subunit)
CIV-MTCO1: Mitochondrially encoded cytochrome c oxidase I (Complex IV subunit)
DDIT4: DNA damage-inducible transcript 4
DEFB4: Defensin beta 4
DMSO: Dimethyl sulfoxide
ECAR: Extracellular acidification rate
EGF: Epidermal growth factor
ECL: Enhanced chemiluminescence
FCCP: Carbonyl cyanide-p-trifluoromethoxyphenylhydrazone
FKBP12: FK506-Binding Protein 12 kDa
FRB: FKBP12-rapamycin binding domain
Glut1: Glucose transporter 1
H&E: Hematoxylin and eosin
TNF-α: Tumor Necrosis Factor-alpha
IL: Interleukin
IMQ: Imiquimod
i.p.: Intraperitoneal
KSFM: Keratinocyte serum-free medium
LCN2: Lipocalin 2
LN: Lymph node
mTOR: Mechanistic target of rapamycin
mTORC1: Mechanistic target of rapamycin complex 1
MTT: 3-(4,5-dimethylthiazol-2-yl)-2,5-diphenyltetrazolium bromide
OCR: Oxygen consumption rate
OXPHOS: Oxidative phosphorylation
P70S6K: p70 ribosomal S6 kinase
PASI: Psoriasis Area and Severity Index
PBMCs: Peripheral blood mononuclear cells
PMA: Phorbol 12-myristate 13-acetate
qPCR: Quantitative real-time polymerase chain reaction
Rapa: Rapamycin
Raptor: Regulatory-associated protein of mTOR
ROS: Reactive oxygen species
Rot/AA: Rotenone/Antimycin A
siCtrl: Control small interfering RNA
siDDIT4: DDIT4-targeting small interfering RNA
TPA: 12-O-tetradecanoylphorbol-13-acetate
GLUT1: Glucose transporter 1
MitoTracker: mitochondrial membrane potential (Δψm)
MitoSOX: mitochondria-specific ROS
CMXROS: a Δψm-dependent mitochondrial dye
Th1: T helper type 1 cell
Th17: T helper type 17 cell
Teff: effector T cell
Treg: regulatory T cell
CETSA: Cellular thermal shift assay
