# Supplementary material for: Nomilin Rewires mTOR-Driven Immunometabolism via DDIT4 to Restrain Psoriasis
Source: Int J Biol Sci. 2026 Sep 1;22(14):7512–32. doi: 10.7150/ijbs.133330 (PMC13617650; doi:10.7150/ijbs.133330)
Supplement: Supplementary file 1 [file ijbsv22p7512s1.pdf]

Supplementary Materials

Nomilin Rewires mTOR-Driven Immunometabolism via DDIT4 to Restrain Psoriasis

Ha Eun Kim, Seung Taek Lee, Hee-Suk Park, Hyun-Su Lee, Jong Yeong Lee, Dae-Ki Kim, Si-Gyun Roh, Kyung-Hwa Nam, Sang-Hyun Kim, Jin Kyeong Choi

Corresponding author: Jin Kyeong Choi

E-mail: jkchoi@jbnu.ac.kr

Files include:

Materials and Methods

Table S1 to S4

Figures S1 and S6

References

Materials and Methods

TPA-induced psoriasis-like mouse model

Male C57BL/6 mice were randomly assigned to the indicated experimental groups (*n* = 5 mice per group). Psoriasis-like dermatitis was induced by topical application of 12-O-tetradecanoylphorbol-13-acetate (TPA, Sigma-Aldrich) (2.5 µg/ear/day), dissolved in acetone, to both ears from day 0 to day 6, as previously described [1]. Nomilin was administered intraperitoneally at low (200 µg per mouse) and high (400 µg per mouse) doses from day 1 to day 6. Rapamycin (600 µg per mouse) was used as a positive control. Treatments were administered once daily for six consecutive days. Ear thickness was measured daily at the same time using a Mitutoyo dial thickness gauge (Mitutoyo Co., Tokyo, Japan). On day 7, mice were euthanized using CO_2, and ear tissues were harvested. Samples were divided for histology, qPCR, Western blotting, flow cytometry, Seahorse analysis, and mitochondrial assays.

Isolation of Primary Human Keratinocytes

Discarded skin specimens were obtained from adult donors undergoing elective plastic surgery procedures at Jeonbuk National University Hospital. Upon collection, skin samples were rinsed twice with phosphate-buffered saline (PBS; Welgene, Gyeongsan, Republic of Korea; Cat#LB204-02) containing 1% penicillin–streptomycin (Gibco, Thermo Fisher Scientific, Waltham, MA, USA; Cat#15140-122) to remove blood and debris. Subcutaneous fat was carefully removed, and the tissue was cut into approximately 1 cm^2 pieces with the epidermal surface facing downward. Tissue fragments were incubated in PBS containing Dispase II (2.4 mg/mL; Roche Diagnostics, Mannheim,

Germany; Cat#04942078001) at 4°C for approximately 20 h to allow separation of the epidermis from the dermis. The following day, the epidermal sheets were gently detached from the dermis using sterile forceps and transferred into 15 mL conical tubes containing 5 mL of 0.05% trypsin-EDTA (Gibco, Thermo Fisher Scientific, Waltham, MA, USA; Cat#25300-054). Samples were incubated at 37°C for 10 min to dissociate keratinocytes. The enzymatic reaction was terminated by adding an equal volume of Keratinocyte Serum-Free Medium (KSFM; Gibco, Thermo Fisher Scientific, Waltham, MA, USA; Cat#17005-042) supplemented with epidermal growth factor (EGF), bovine pituitary extract (BPE), and gentamicin (50 μg/mL; Gibco, Thermo Fisher Scientific, Waltham, MA, USA; Cat#15750-060). The epidermal sheets were mechanically triturated to release individual keratinocytes. The resulting cell suspension was filtered through a 70 μm cell strainer (SPL Life Sciences, Pocheon-si, Gyeonggi-do, Republic of Korea; Cat#93070) into a 50 mL conical tube and centrifuged at 180 × g for 10 min. The cell pellet was resuspended in KSFM supplemented with the appropriate media and seeded onto collagen type IV-coated culture dishes (Human Collagen Type IV; Sigma-Aldrich, St. Louis, MO, USA; Cat#C5533) prepared according to the manufacturer's instructions. Cells were maintained under standard culture conditions (37°C in a humidified incubator with 5% CO_2) and were used between passages 2 and 4 for all experiments.

Measurement of mitochondrial mass and ROS

Primary human keratinocytes and single cells isolated from mouse ear tissues (1×10^5 cells/well) were seeded in 96-well U-bottom plates. Following the indicated treatments, cells were incubated with MitoTracker Green FM (0.2 μM; Thermo Fisher Scientific), MitoTracker Red CMXROS (0.2 μM; Thermo Fisher Scientific), or MitoSOX Red (1 μM; Thermo Fisher Scientific) for 1 h at 37°C in the

dark. MitoTracker Green FM was used to assess mitochondrial mass independent of membrane potential, MitoTracker Red CMXROS was used to evaluate mitochondrial membrane potential-dependent staining, and MitoSOX Red was used to measure mitochondrial superoxide production. After incubation, cells were washed once with pre-warmed medium and stained with Live/Dead Fixable Dead Cell dye (Invitrogen) for 20 min at room temperature in the dark to exclude non-viable cells. Fluorescence intensity was measured using an Attune NxT acoustic focusing cytometer (Thermo Fisher Scientific), and data were analyzed using FlowJo software.

Transmission Electron Microscopy (TEM)

Human keratinocytes (1×10^7 cells) were stimulated with IL-17A in the presence or absence of Nomilin for 24 h. Following treatment, cells were detached using 0.25% trypsin-EDTA, collected by centrifugation, and transferred into 1.5-mL microcentrifuge tubes. All subsequent TEM sample preparation procedures were performed in the tubes. Cell pellets were fixed with 2% paraformaldehyde (Electron Microscopy Sciences, Hatfield, PA, USA; Cat#15713) and 2% glutaraldehyde (Electron Microscopy Sciences, Hatfield, PA, USA; Cat#16400) in 0.05 M sodium cacodylate buffer (Electron Microscopy Sciences, Hatfield, PA, USA; Cat#11654, pH 7.2) at 4°C overnight. After primary fixation, the samples were washed three times for 10 min each with 0.05 M sodium cacodylate buffer (pH 7.2) at 4°C. The specimens were post-fixed with 1% osmium tetroxide (Electron Microscopy Sciences, Hatfield, PA, USA; Cat#19192) in 0.05 M sodium cacodylate buffer (pH 7.2) for 1.5 h at 4°C, followed by two washes with distilled water for 10 min each at room temperature. The samples were then subjected to en bloc staining with 0.5% aqueous uranyl acetate (Electron Microscopy Sciences, Hatfield, PA, USA; Cat#22400) at 4°C overnight. The specimens were dehydrated through a graded ethanol series (40%, 50%, 70%, 80%, 90%, 100%, 100%, 100%) for 10 min at room temperature at

each step and subsequently treated with 100% propylene oxide (Electron Microscopy Sciences, Hatfield, PA, USA; Cat#20401) twice for 15 min each at room temperature. Resin infiltration was performed using a 1:1 mixture of propylene oxide and EMbed 812 resin kit (Electron Microscopy Sciences, Hatfield, PA, USA; Cat#14120) for 2 h, followed by a 1:3 mixture of propylene oxide and EMbed 812 resin overnight at room temperature. The samples were subsequently infiltrated with 100% EMbed 812 resin for 2 h and polymerized at 60°C for 48 h. The EMbed 812 resin mixture consisted of 20 mL EMbed 812 resin, 16 mL dodecenyl succinic anhydride (DDSA), 8 mL nadic methyl anhydride (NMA), and 0.88 mL 2,4,6-tris(dimethylaminomethyl)phenol (DMP-30). Polymerized blocks were trimmed, and 70 nm ultrathin sections were prepared using a 45° General Purpose diamond knife mounted on an ultramicrotome (Leica UC7, Leica Microsystems, Wetzlar, Germany). Sections were mounted on 200 mesh copper grids (Electron Microscopy Sciences, Hatfield, PA, USA; Cat#78138-01) and examined using a transmission electron microscope equipped with a LaB6 electron source operated at an accelerating voltage of 120 kV. Digital images were acquired using a Gatan Rio9 CMOS camera (Gatan Inc., Pleasanton, CA, USA).

Cellular thermal shift assay (CETSA)

Cellular Thermal Shift Assay (CETSA) was performed to evaluate the thermal stabilization of target proteins upon HaCaT cells were seeded at a density of 1×10^6 cells/well in 6 well plates and cultured overnight. Cells were treated with Nomilin for 12 h, whereas vehicle-treated cells served as controls. Cells were washed once with ice-cold PBS, harvested by scraping, and resuspended in PBS. Equal volumes of the cell suspension were aliquoted into PCR tubes and heated at 37, 45, 50, 55, 60, 65, and 70°C for 3 min using a thermal cycler. Immediately after heat treatment, the samples were rapidly frozen in liquid nitrogen. NP-40 lysis buffer supplemented with a protease inhibitor cocktail was then

added, followed by three freeze–thaw cycles using liquid nitrogen and room-temperature thawing to ensure complete cell lysis. The lysates were centrifuged at 20,000 × g for 20 min at 4°C, and the soluble supernatants were collected for protein quantification and Western blot analysis. Densitometric analysis of immunoblot bands was performed using ImageJ software (National Institutes of Health, Bethesda, MD, USA). Protein band intensities at each temperature were normalized to the corresponding signal at 37°C, which was set to 100% soluble protein. The normalized data were fitted using a four-parameter logistic (4PL) nonlinear regression model in GraphPad Prism 9 (GraphPad Software, San Diego, CA, USA), and the melting temperature (Tm) was calculated as the midpoint of the fitted thermal denaturation curve. The change in melting temperature (ΔTm) was determined by comparing the Tm values of Nomilin-treated and vehicle-treated samples.

Pull-down Assay

To evaluate the direct interaction between nomilin and mTOR, nomilin-conjugated Sepharose 4B beads were generated as previously described with minor modifications [2]. Briefly, CNBr-activated Sepharose 4B beads were hydrated in 1 mM HCl and coupled to nomilin dissolved in DMSO in coupling buffer (0.1 M $NaHCO_3$, pH 8.3, containing 0.5 M NaCl). Following overnight incubation at 4°C, the beads were blocked with 0.1 M Tris-HCl (pH 8.0) and sequentially washed with acetate buffer (0.1 M sodium acetate, pH 4.0, containing 0.5 M NaCl) and coupling buffer to remove unbound compounds. Cell lysates were incubated overnight at 4°C with control Sepharose 4B beads or nomilin-conjugated Sepharose 4B beads in reaction buffer (50 mM Tris-HCl, 5 mM EDTA, 150 mM NaCl, 1 mM DTT, 0.01% NP-40, and protease inhibitor cocktail). For competition assays, increasing concentrations of free nomilin were added during incubation. After extensive washing, bound proteins

were eluted in SDS sample buffer, separated by SDS-PAGE, and analyzed by Western blotting using antibodies against mTOR. The intensity of precipitated mTOR was quantified by densitometric analysis and normalized to input controls.

Cell viability assay

Cell viability was evaluated using thiazolyl blue tetrazolium bromide (MTT) (Sigma-Aldrich, St. Louis, MO, USA; Cat#M2128) and Cell Counting Kit-8 (CCK-8) (Dojindo Laboratories, Kumamoto, Japan; Cat#HN741) assays according to the manufacturers' instructions. For the MTT assay, primary human keratinocytes were seeded in 96 well plates, incubated overnight, and treated under the indicated experimental conditions for 24 h. Subsequently, MTT solution (0.5 mg/mL in PBS) was added to each well at 20% of the culture medium volume and incubated for 2-4 h at 37°C in the dark. The culture medium was then removed, and the resulting formazan crystals were dissolved in 100 μL dimethyl sulfoxide (DMSO) per well. Absorbance was measured at 570 nm using a microplate reader. For the CCK-8 assay, $CD4^+$ T cells and human peripheral blood mononuclear cells (PBMCs) were cultured in 96 well plates containing 100 μL medium per well and treated under the indicated experimental conditions for 48 h and 72 h, respectively. Subsequently, 10 μL of CCK-8 solution was added to each well and incubated for 2–4 h at 37°C. Absorbance was measured at 450 nm using a microplate reader, and cell viability was expressed as a percentage relative to the untreated control group.

In vitro Th17 differentiation

Naive $CD4^+CD62L^+$ T cells were isolated from the spleens and peripheral lymph nodes of C57BL/6

mice using the $CD4^+CD62L^+$ T Cell Isolation Kit II (Miltenyi Biotec, Bergisch Gladbach, Germany; Cat#130-106-643) according to the manufacturer's instructions. For Th17 differentiation, 48 well plates were coated overnight at 4°C with anti-mouse CD3 antibody (Bio X Cell, Lebanon, NH, USA; Cat#BE0002; 2 μg/mL in PBS, 300 μL/well) and washed twice with PBS before cell seeding. Purified naive $CD4^+CD62L^+$ T cells were seeded at 1.5×10^6 cells/well in TexMACS medium (Miltenyi Biotec, Bergisch Gladbach, Germany; Cat#130-097-196) supplemented with 10% FBS and 1% penicillin-streptomycin. Cells were stimulated with soluble anti-CD28 antibody (BD Biosciences, San Jose, CA, USA; Cat#553294; 2 μg/mL) and cultured under Th17-polarizing conditions in the presence of anti-IFN-γ antibody (BioLegend, San Diego, CA, USA; Cat#505834; 50 μg/mL), anti-IL-4 antibody (BioLegend, San Diego, CA, USA; Cat#504122; 50 μg/mL), anti-IL-2 antibody (BioLegend, San Diego, CA, USA; Cat#503808; 50 μg/mL), recombinant mouse IL-6 (BioLegend, San Diego, CA, USA; Cat#575702; 20 ng/mL), and recombinant human TGF-β1 (PeproTech, Rocky Hill, NJ, USA; Cat#100-21; 2 ng/mL). On day 2, cells were treated with Nomilin. On day 4, half of the culture medium was replaced with fresh medium supplemented with cytokines and neutralizing antibodies at half of their initial concentrations. Cells were maintained under Th17-polarizing conditions for a total of 6 days and harvested on day 6 for flow cytometric and Western blot analyses.

Table S1. Enrolled psoriasis patient information

Patient ID	Sex	Age	Severity	PASI
1	Male	59	Severe	10.2
2	Female	60	Mild	3.3
3	Female	35	Severe	20.7
4	Female	43	Severe	10.6
5	Male	33	Mild	3.1
6	Male	57	Severe	22.5
7	Female	25	Moderate	8.2
8	Female	40	Moderate	7.6
9	Female	44	Moderate	7
10	Male	54	Severe	13.7
11	Male	31	Severe	11.8
12	Male	59	Moderate	7.8
13	Male	37	Mild	3.1
14	Male	51	Severe	10.7
15	Female	58	Moderate	8.6

16	Female	67	Moderate	5.1
17	Male	56	Moderate	9.3
18	Female	51	Mild	4.4
19	Male	51	Severe	21.6
20	Female	25	Moderate	7.3
21	Female	36	Severe	13.8

Abbreviation: PASI, Psoriasis Area and Severity Index.

Disease severity definition: Mild, PASI < 10; Severe, PASI ≥ 10.

Inclusion criteria:

1. Patients diagnosed with plaque psoriasis by a board-certified dermatologist, with a disease duration of ≥3 months.
2. Age between 18 and 70 years at the time of enrollment.
3. Provision of written informed consent prior to participation.

Exclusion criteria:

1. Age <18 years or >70 years.
2. Presence of autoimmune or other systemic diseases within the previous three months.
3. Evidence of active infection within two weeks prior to enrollment.
4. Use of systemic medications for conditions other than psoriasis (including oral corticosteroids, immunosuppressive agents, antibiotics, biologic agents, or anticancer drugs) within the previous three months.

5. History of substance abuse, alcohol dependence, or psychiatric disorders within the previous three months.

Table S2. Forward and reverse primer sequence

Human

Gene	Forward primer (5'>3')	Reverse primer (5'>3')
Il1β	GCT GAT GGC CCT AAA CAG ATG AA	TGA AGC CCT TGC TGT AGT GGT G
Il6	CCC CTG ACC CAA CCA CAA AT	CAT TTG CCG AAG AGC CCT CA
Defb4	TGATGCCTCTTCCAGGTGTT	GCCTCCTCATGGCTTTTTGC
Lcn2	CCACCTCAGACCTGATCCCA	CCCCTGGAATTGGTTGTCCTG
S100a7	ACGTGATGACAAGATTGACAAGC	GCGAGGTAATTTGTGCCCTTT
S100a8	AGACCGAGACCGAGTGTCCTC	TGCCACGCCCATCTTTAT
S100a9	GGTCATAGAACACATCATGGAGG	GGCCTGGCTTATGGTGGTG
β-actin	AGAGCTACGAGCTGCCTGAC	AGCACTGTGTTGGCGTACAG

Mouse

Gene	Forward primer (5'>3')	Reverse primer (5'>3')
Tnfa	GGC AGG TCT ACT TTG GAG TCA TTG C	ACA TTC GAG GCT CCA GTG AAT TCG G
Il1β	CCA AAA GAT GAA GGG CTG CTT	TGC TGC TGC GAG ATT TGA AG
Lcn2	TCC AGT TGC CTT CTT GGG AC	GT CTG TTG GGA GTG GTA TC
S100a7	CCC TGC ACC AAG AGC AAC	GCACAGTTTTGTGGGGTTTT
S100a8	ATC ACC ATG CCC TCT ACA AGA ATG	GTC CAA TTC TCT GAA CAA GTT TTC G
S100a9	CAC CCT GAG CAA GAA GGA AT	TGT CAT TTA TGA GGG CTT CAT TT
Il17a	CTCAAAGCTCAGCGTGTCCAAACA	TATCAGGGTCTTCATTGCGGTGGA

Il17E	CCCCTGGAGATATGAGTTGGAC	GTCTGTAGGCTGACGCAGTG
Il17F	CAGGAAGACAGCACCATGAA	TCTTCTCCAACCTGAAGGAATTA G
Il22	AT GAG AGA GCG CTG CTA CCT GG	AAGGACGCCACCTCCTGCAT GT
Glut1	ACT GGG CAA GTC CTT TGA G	CAT GAT GGA GTC TAA GCC AAA C
β-actin	ACC CTA AGG CCA ACC GTG AA	ATG GCG TGA GGG AGA GCA TAG

Table S3. Antibodies used for Western blot analysis

Target Protein	Company	Catalog No.	Dilution
p-mTOR (Ser2448)	Cell Signaling Technology	#5536	1:1000
mTOR	Cell Signaling Technology	#2983	1:1000
p-Raptor (Ser792)	Cell Signaling Technology	#2083	1:1000
Raptor	Cell Signaling Technology	#2280	1:1000
p-P70S6K(Thr389)	Cell Signaling Technology	#9234	1:1000
P70S6K	Cell Signaling Technology	#9202	1:1000
p-4EBP1 (Thr37/46)	Cell Signaling Technology	#2855	1:1000
4EBP1	Cell Signaling Technology	#9644	1:1000
p-AMPK (Thr172)	Cell Signaling Technology	#2535	1:1000
AMPK	Cell Signaling Technology	#8531	1:1000
DDIT4	Proteintech	10638-1-AP	1:1000
β-actin	Cell Signaling Technology	#3700	1:5000
HRP-conjugated anti-rabbit IgG	Enzo Life Sciences	ADI-SAB-300	1:10000

Table S4. Antibodies used for flow cytometric analysis

Species	Marker	Fluorochrome	Company	Cat. No.	Clone	Country
Mouse	CD4	BV421	BioLegend	100544	RM4-5	San Diego, USA
Mouse	CD25	BV510	BioLegend	102041	PC61	San Diego, USA
Mouse	IFN-γ	FITC	BD Biosciences	554411	XMG1.2	Franklin Lakes, USA
Mouse	IL-17A	PerCP-Cy5.5	BD Biosciences	560666	TC11-18H10	Franklin Lakes, USA
Mouse	IL-22	APC	Invitrogen	17-7222-82	IL22JOP	Carlsbad, USA
Mouse	Foxp3	PE-Cy7	Invitrogen	25-5773-82	FJK-16s	Carlsbad, USA
Mouse	RORγt	PE	Invitrogen	12-6988-82	AFKJS-9	Carlsbad, USA
Mouse	CD11b	FITC	BioLegend	101206	M1/70	San Diego, USA
Mouse	F4/80	PE	BioLegend	123110	BM8	San Diego, USA
Mouse	Ly6C	PerCP-Cy5.5	BD Biosciences	560525	AL-21	Franklin Lakes, USA
Mouse	Ly6G	BV510	BD Biosciences	740157	1A8	Franklin Lakes, USA
Mouse	CD86	BV421	BioLegend	105123	PO3	San Diego, USA
Mouse	CD163	PE-Cy7	BioLegend	155320	S150491	San Diego, USA
Human	CD4	BV421	BioLegend	344632	SK3	San Diego, USA
Human	CD25	BV510	BD Biosciences	563351	M-A251	Franklin Lakes, USA
Human	TNF-α	FITC	BioLegend	502906	MAb11	San Diego, USA

Human	IFN-γ	PerCP-Cy5.5	BD Biosciences	560742	4S.B3	Franklin Lakes, USA
Human	IL-17A	APC	BD Biosciences	560490	N49-653	Franklin Lakes, USA
Human	Foxp3	PE	Invitrogen	12-4776-42	PCH101	Carlsbad, USA
Mouse/ Human	Fixable Viability Dye eFluor™ 780	APC-Cy7	Invitrogen	65-0865-14	-	Carlsbad, USA

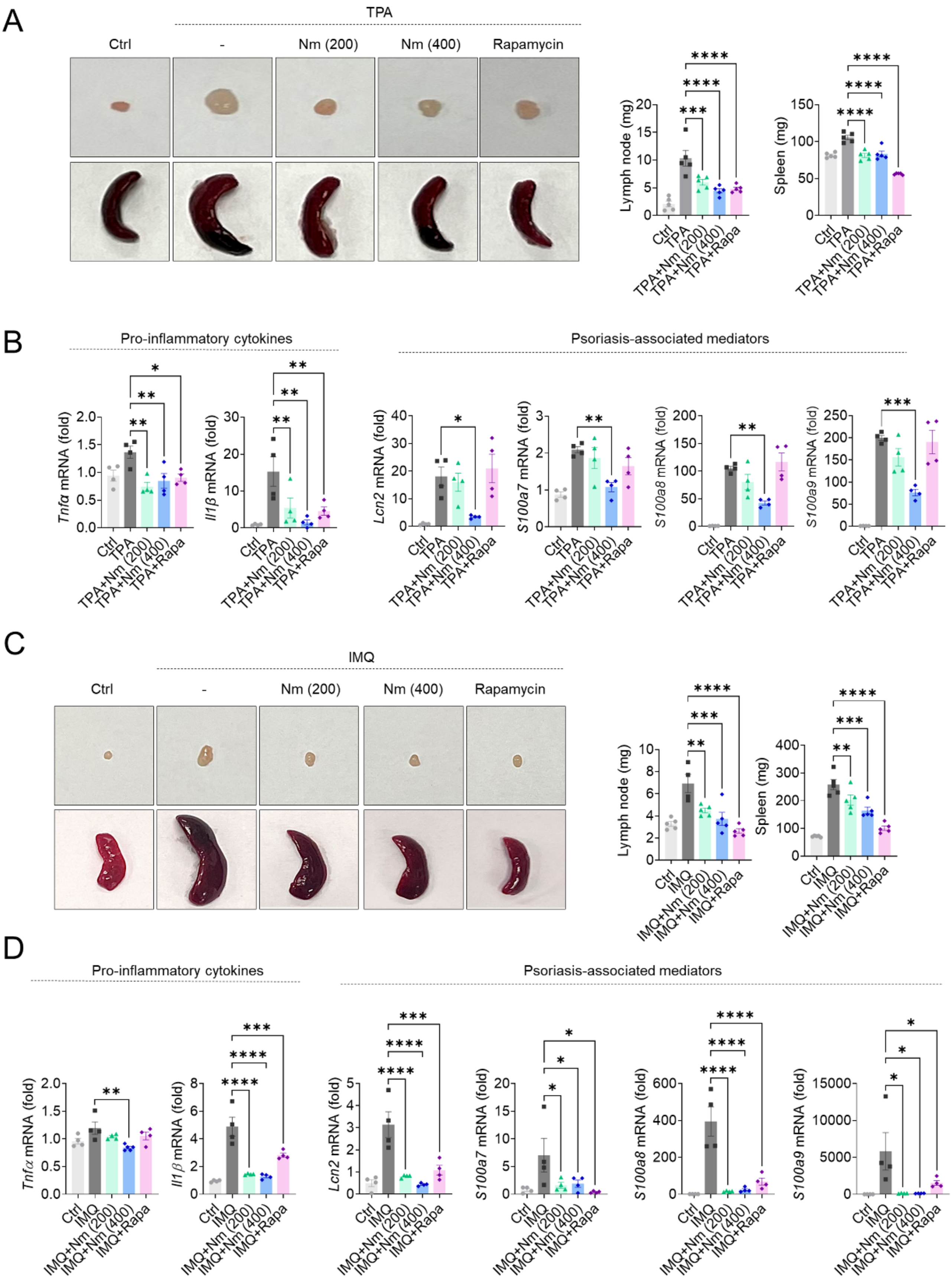
A
TPA
Ctrl
-
Nm (200)
Nm (400)
Rapamycin
Lymph node (mg)
Spleen (mg)
Ctrl
TPA
TPA+Nm (200)
TPA+Nm (400)
TPA+Rapa
B
Pro-inflammatory cytokines
Psoriasis-associated mediators
Tnfα mRNA (fold)
Il1β mRNA (fold)
Lcn2 mRNA (fold)
S100a7 mRNA (fold)
S100a8 mRNA (fold)
S100a9 mRNA (fold)
C
IMQ
Ctrl
-
Nm (200)
Nm (400)
Rapamycin
Lymph node (mg)
Spleen (mg)
Ctrl
IMQ
IMQ+Nm (200)
IMQ+Nm (400)
IMQ+Rapa
D
Pro-inflammatory cytokines
Psoriasis-associated mediators
Tnfα mRNA (fold)
Il1β mRNA (fold)
Lcn2 mRNA (fold)
S100a7 mRNA (fold)
S100a8 mRNA (fold)
S100a9 mRNA (fold)

Supplementary Figure S1. Nomilin suppresses systemic inflammatory responses and psoriasis-associated gene expression in TPA- and IMQ-induced psoriasiform inflammation. (A) Representative images and quantification of draining lymph nodes and spleens collected from mice subjected to the TPA-induced psoriasiform dermatitis model. Nomilin (Nm; 200 or 400 μg per mouse) or rapamycin was administered as described in Fig. 1B. **(B)** Relative mRNA expression levels of pro-inflammatory cytokines (*Tnfα* and *Il1β*) and psoriasis-associated mediators (*Lcn2*, *S100a7*, *S100a8*, and *S100a9*) in TPA-induced ear lesions, determined by quantitative PCR and expressed relative to the control group. **(C)** Representative images and quantification of draining lymph nodes and spleens collected from mice subjected to the IMQ-induced psoriasis-like skin inflammation model. Nomilin (Nm; 200 or 400 μg per mouse) or rapamycin was administered as described in Fig. 1H. **(D)** Relative mRNA expression levels of pro-inflammatory cytokines (*Tnfα* and *Il1β*) and psoriasis-associated mediators (*Lcn2*, *S100a7*, *S100a8*, and *S100a9*) in IMQ-induced skin lesions, determined by quantitative PCR and expressed relative to the control group. Data are presented as mean ± SEM (n = 4-5 mice per group). Statistical significance was determined by one-way ANOVA followed by Holm-Šídák multiple-comparison test. ****P < 0.0001, ***P < 0.001, **P < 0.01, *P < 0.05.

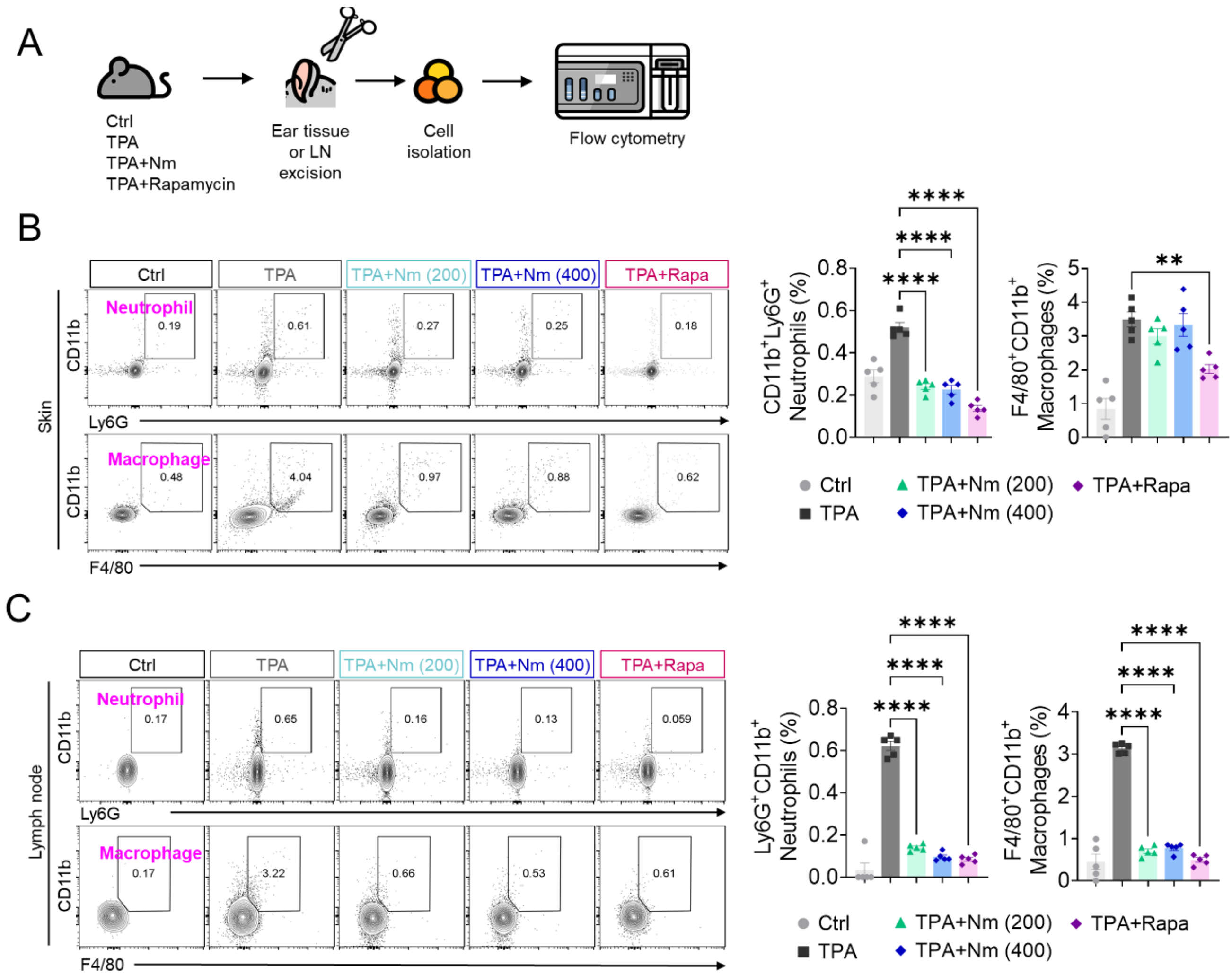
A
Ctrl
TPA
TPA+Nm
TPA+Rapamycin
Ear tissue or LN excision
Cell isolation
Flow cytometry
B
Ctrl
TPA
TPA+Nm (200)
TPA+Nm (400)
TPA+Rapa
Neutrophil
0.19
0.61
0.27
0.25
0.18
Macrophage
0.48
4.04
0.97
0.88
0.62
Skin
CD11b
Ly6G
F4/80
CD11b⁺Ly6G⁺ Neutrophils (%)
F4/80⁺CD11b⁺ Macrophages (%)
****
**
Ctrl
TPA
TPA+Nm (200)
TPA+Nm (400)
TPA+Rapa
C
Neutrophil
0.17
0.65
0.16
0.13
0.059
Macrophage
0.17
3.22
0.66
0.53
0.61
Lymph node
Ly6G⁺CD11b⁺ Neutrophils (%)
F4/80⁺CD11b⁺ Macrophages (%)

D

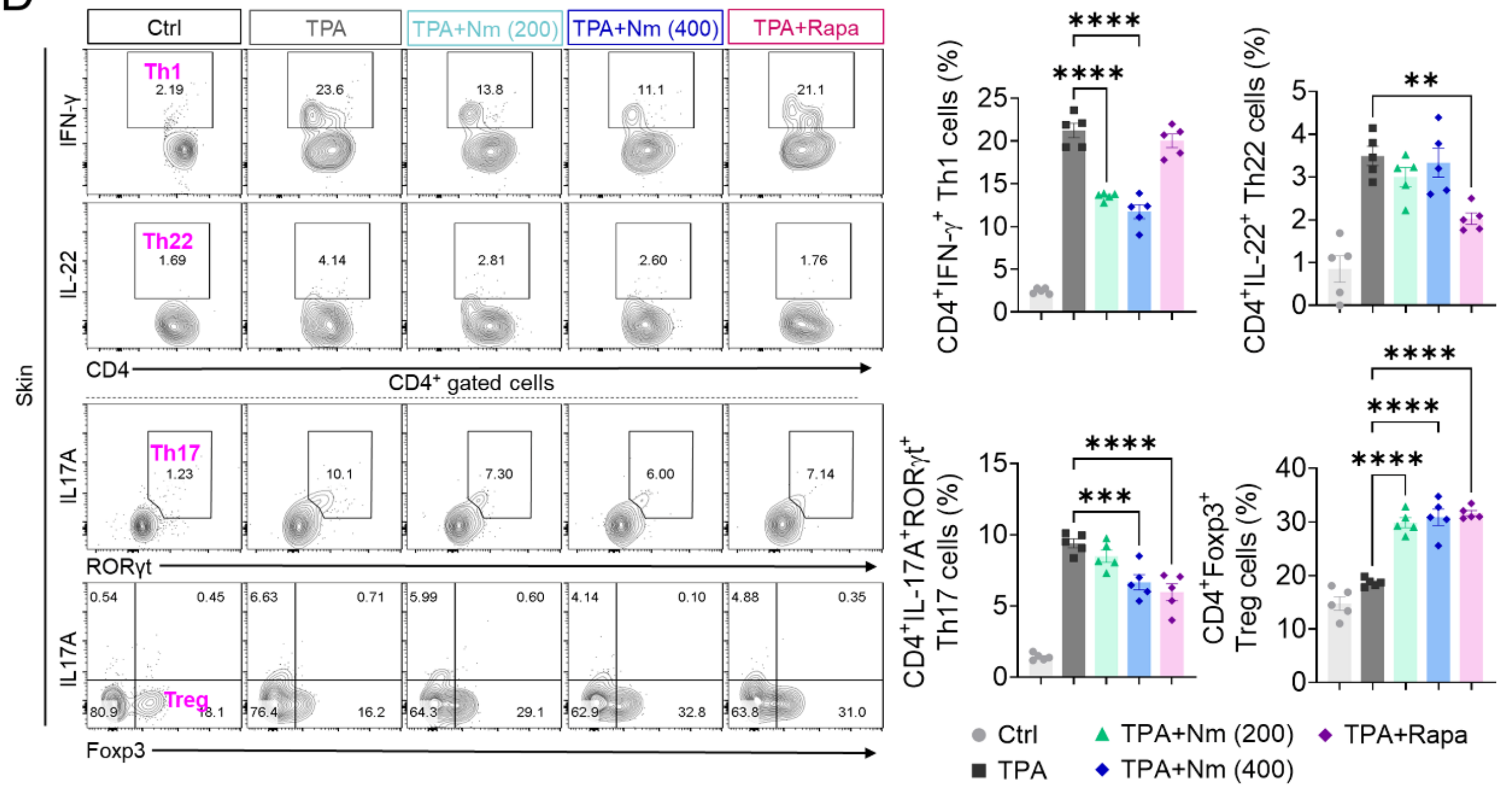

Ctrl
TPA
TPA+Nm (200)
TPA+Nm (400)
TPA+Rapa
Skin
Th1
Th22
Th17
Treg
IFN-γ
IL-22
IL17A
CD4
RORγt
Foxp3
CD4⁺ gated cells
CD4⁺IFN-γ⁺ Th1 cells (%)
CD4⁺IL-22⁺ Th22 cells (%)
CD4⁺IL-17A⁺RORγt⁺ Th17 cells (%)
CD4⁺Foxp3⁺ Treg cells (%)

E

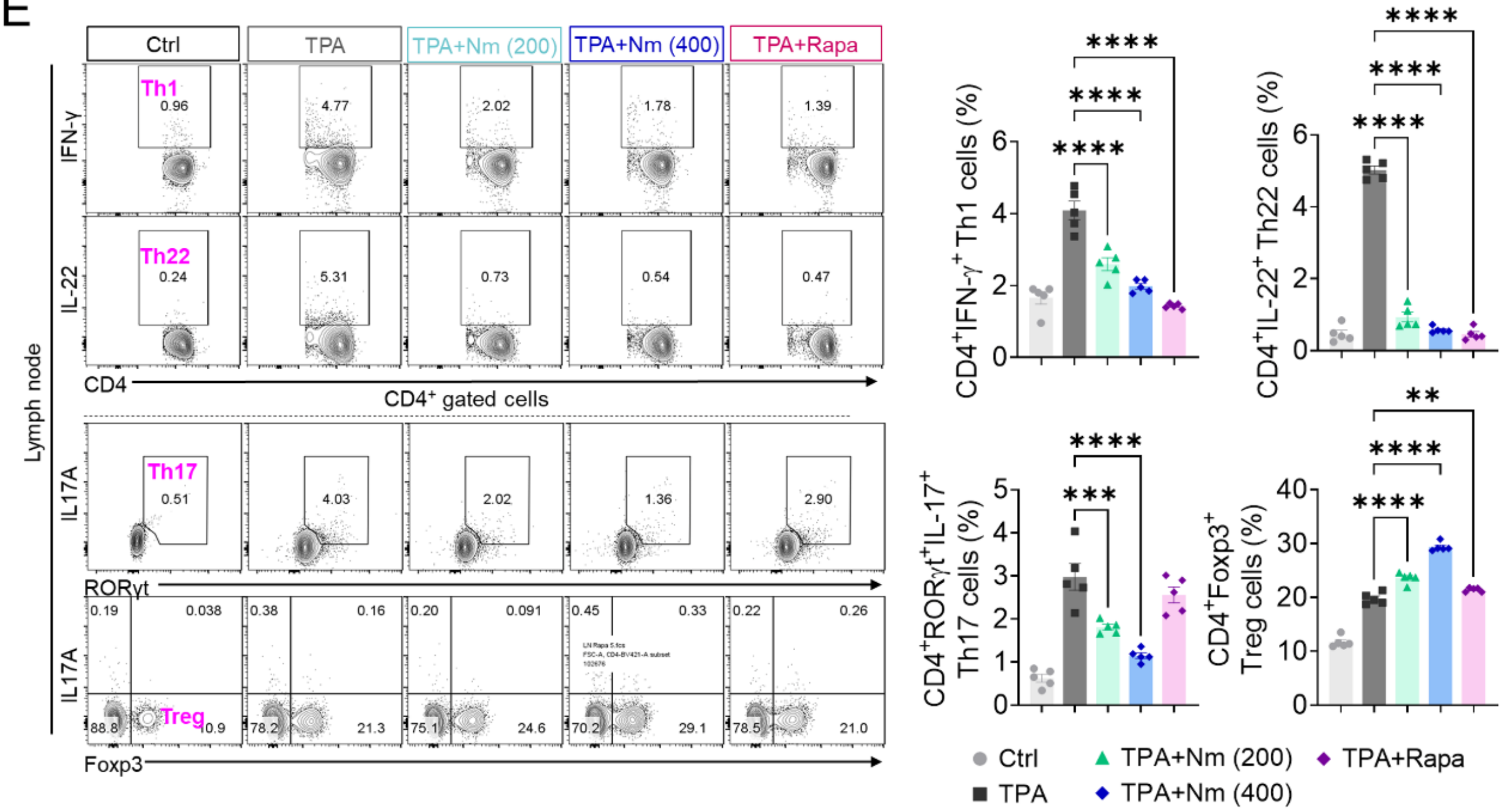

Ctrl
TPA
TPA+Nm (200)
TPA+Nm (400)
TPA+Rapa
Lymph node
Th1
Th22
Th17
Treg
IFN-γ
IL-22
IL17A
CD4
RORγt
Foxp3
CD4⁺ gated cells
CD4⁺IFN-γ⁺ Th1 cells (%)
CD4⁺IL-22⁺ Th22 cells (%)
CD4⁺RORγt⁺IL-17⁺ Th17 cells (%)
CD4⁺Foxp3⁺ Treg cells (%)

F

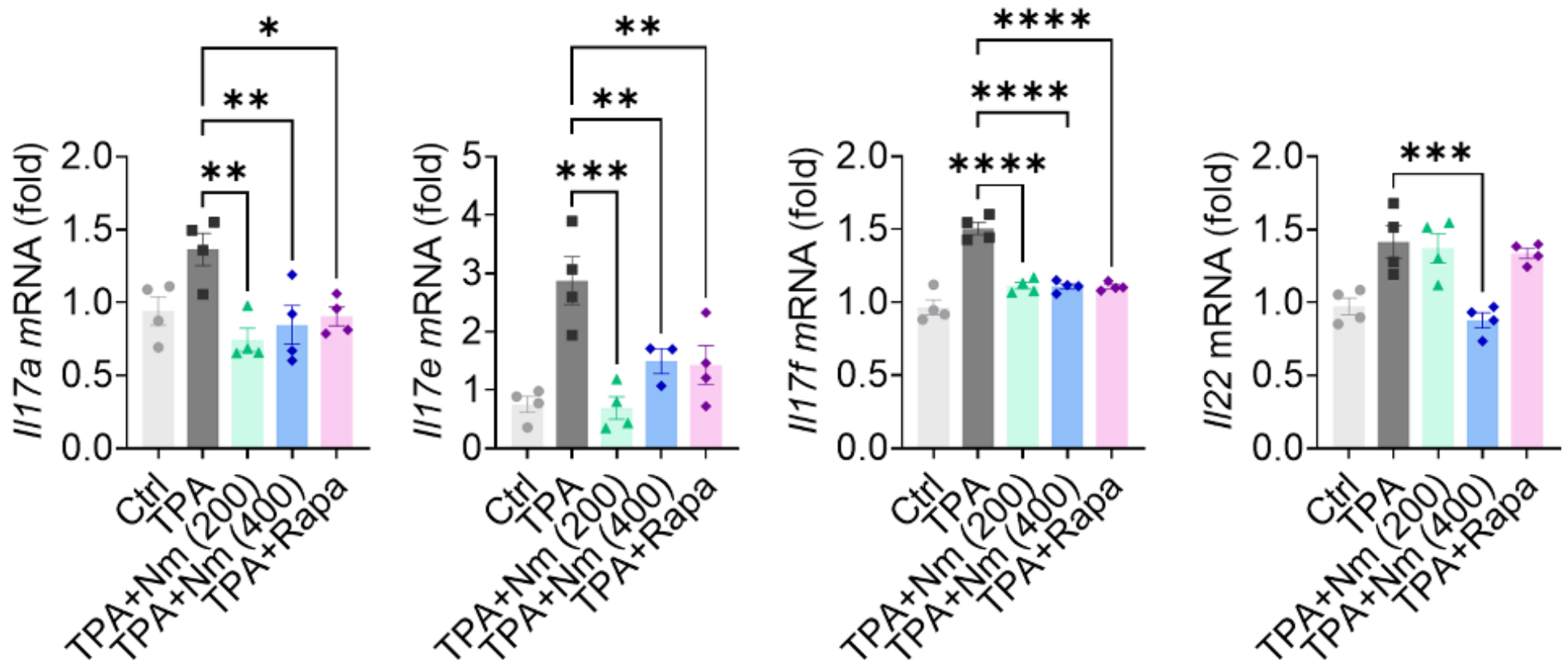

Il17a mRNA (fold)
Il17e mRNA (fold)
Il17f mRNA (fold)
Il22 mRNA (fold)
Ctrl
TPA
TPA+Nm (200)
TPA+Nm (400)
TPA+Rapa

Supplementary Figure S2. Nomilin modulates innate and adaptive immune cell responses in TPA-induced psoriasiform inflammation. (A) Experimental scheme for flow cytometric analysis of immune cell populations isolated from ear skin and draining lymph nodes of mice subjected to TPA-induced psoriasiform skin lesions. **(B)** Representative flow cytometry plots and quantification of $CD11b^+Ly6G^+$ neutrophils and $CD11b^+F4/80^+$ macrophages in ear skin tissues. **(C)** Representative flow cytometry plots and quantification of $CD11b^+Ly6G^+$ neutrophils and $CD11b^+F4/80^+$ macrophages in draining lymph nodes. **(D)** Representative flow cytometry plots and quantification of skin-infiltrating $CD4^+$ T-cell subsets, including Th1 ($CD4^+IFN\text{-}\gamma^+$), Th22 ($CD4^+IL\text{-}22^+$), Th17 ($CD4^+ROR\gamma t^+IL\text{-}17A^+$), and regulatory T cells ($CD4^+Foxp3^+$). **(E)** Representative flow cytometry plots and quantification of $CD4^+$ T-cell subsets in draining lymph nodes, including Th1, Th22, Th17, and $Foxp3^+$ regulatory T cells. **(F)** Relative mRNA expression levels of Il17a, Il17e, Il17f, and Il22 in TPA-induced skin lesions determined by quantitative PCR and expressed relative to the control group. Data are presented as mean ± SEM (n = 4-5 mice per group). Statistical significance was determined by one-way ANOVA followed by Holm-Šídák multiple-comparison test. ****$P < 0.0001$, ***$P < 0.001$, **$P < 0.01$, *$P < 0.05$.

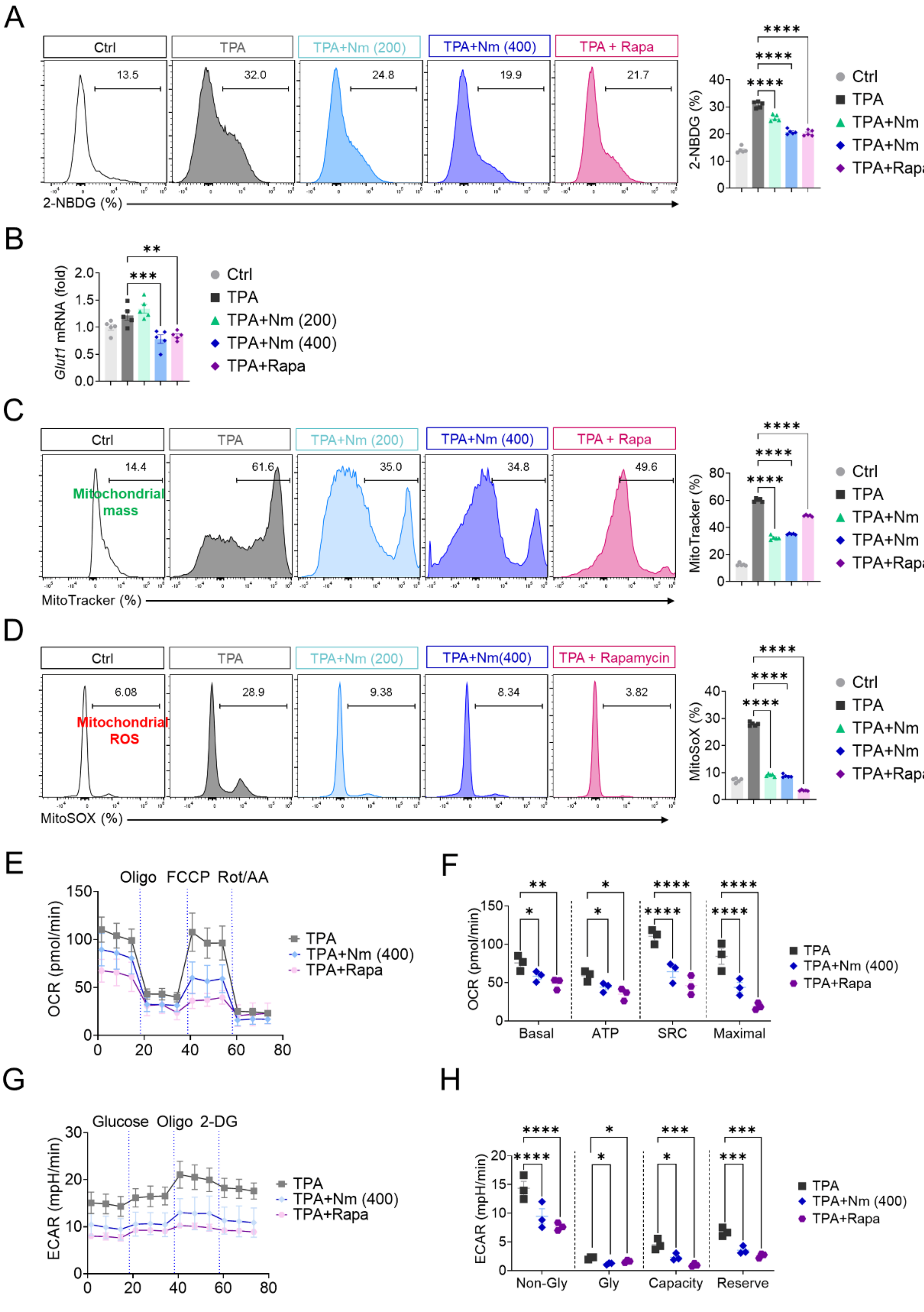

A
Ctrl
TPA
TPA+Nm (200)
TPA+Nm (400)
TPA + Rapa
13.5
32.0
24.8
19.9
21.7
2-NBDG (%)
2-NBDG (%)
****
****
****
Ctrl
TPA
TPA+Nm (200)
TPA+Nm (400)
TPA+Rapa
B
Glut1 mRNA (fold)
**
***
Ctrl
TPA
TPA+Nm (200)
TPA+Nm (400)
TPA+Rapa
C
Ctrl
TPA
TPA+Nm (200)
TPA+Nm (400)
TPA + Rapa
14.4
61.6
35.0
34.8
49.6
Mitochondrial mass
MitoTracker (%)
MitoTracker (%)
****
****
****
Ctrl
TPA
TPA+Nm (200)
TPA+Nm (400)
TPA+Rapa
D
Ctrl
TPA
TPA+Nm (200)
TPA+Nm(400)
TPA + Rapamycin
6.08
28.9
9.38
8.34
3.82
Mitochondrial ROS
MitoSOX (%)
MitoSoX (%)
****
****
****
Ctrl
TPA
TPA+Nm (200)
TPA+Nm (400)
TPA+Rapa
E
Oligo FCCP Rot/AA
OCR (pmol/min)
TPA
TPA+Nm (400)
TPA+Rapa
F
OCR (pmol/min)
Basal
ATP
SRC
Maximal
TPA
TPA+Nm (400)
TPA+Rapa
G
Glucose Oligo 2-DG
ECAR (mpH/min)
TPA
TPA+Nm (400)
TPA+Rapa
H
ECAR (mpH/min)
Non-Gly
Gly
Capacity
Reserve
TPA
TPA+Nm (400)
TPA+Rapa

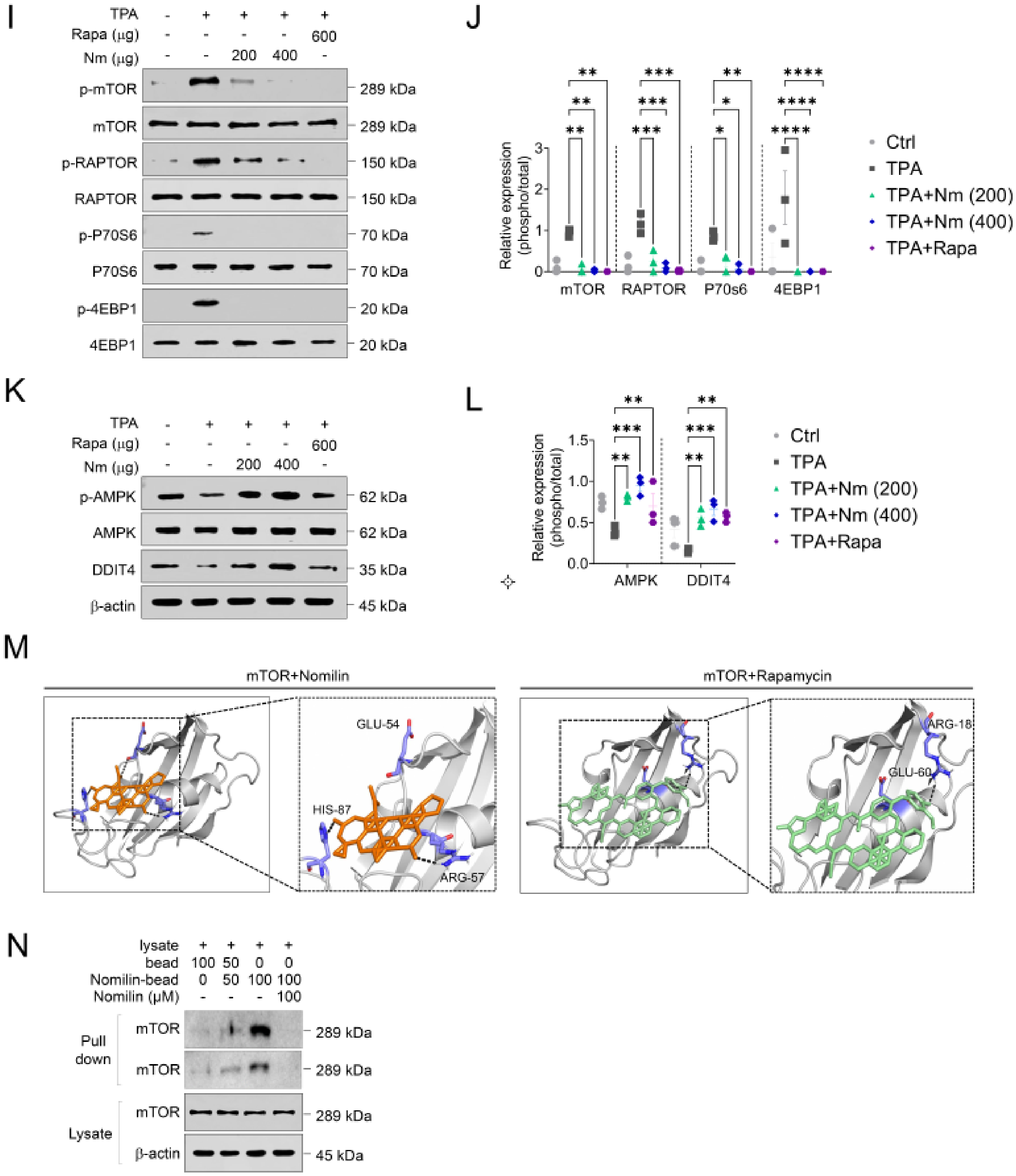

Supplementary Figure 3. Nomilin suppresses metabolic activation and mTORC1 signaling in TPA-induced psoriasiform lesions. (A) Representative histograms and quantification of glucose

uptake measured by 2-NBDG incorporation in cells isolated from TPA-induced ear lesions. **(B)** Relative *Glut1* mRNA expression in ear tissues determined by quantitative PCR and expressed relative to the control group. **(C)** Representative histograms and quantification of mitochondrial mass assessed by MitoTracker staining in cells isolated from TPA-induced skin lesions. **(D)** Representative histograms and quantification of mitochondrial ROS measured by MitoTracker and MitoSOX staining. **(E)** Representative oxygen consumption rate (OCR) profiles obtained by Seahorse extracellular flux analysis. **(F)** Quantification of OCR parameters, including basal respiration, ATP-linked respiration, spare respiratory capacity (SRC), and maximal respiration. **(G)** Representative extracellular acidification rate (ECAR) profiles. **(H)** Quantification of ECAR parameters, including non-glycolytic acidification, glycolysis, glycolytic capacity, and glycolytic reserve. **(I)** Representative Western blot images showing phosphorylation of mTOR and downstream mTORC1 signaling components, including RAPTOR, p70S6K, and 4EBP1, in TPA-induced ear skin lesions. **(J)** Densitometric quantification of phosphorylated mTOR, RAPTOR, p70S6K, and 4EBP1 shown in panel (I), normalized to total protein levels. **(K)** Representative Western blot images showing phosphorylation of AMPK and expression of DDIT4 in TPA-induced ear skin lesions. **(L)** Densitometric quantification of phosphorylated AMPK and DDIT4 expression shown in panel (K). **(M)** Molecular docking models predicting the interaction of nomilin and rapamycin with the FKBP12-associated FRB domain of mTOR. Predicted binding orientations and interacting amino acid residues are shown. **(N)** Pull-down assay using nomilin-conjugated Sepharose beads demonstrating the interaction between nomilin and mTOR. Competition with free nomilin reduced mTOR precipitation, supporting binding specificity. Data are presented as mean ± SEM (n = 4-5 mice per group). Statistical significance was determined by one-way ANOVA followed by Holm-Šídák multiple-comparison test. ****$P < 0.0001$, ***$P < 0.001$, **$P < 0.01$, *$P < 0.05$.

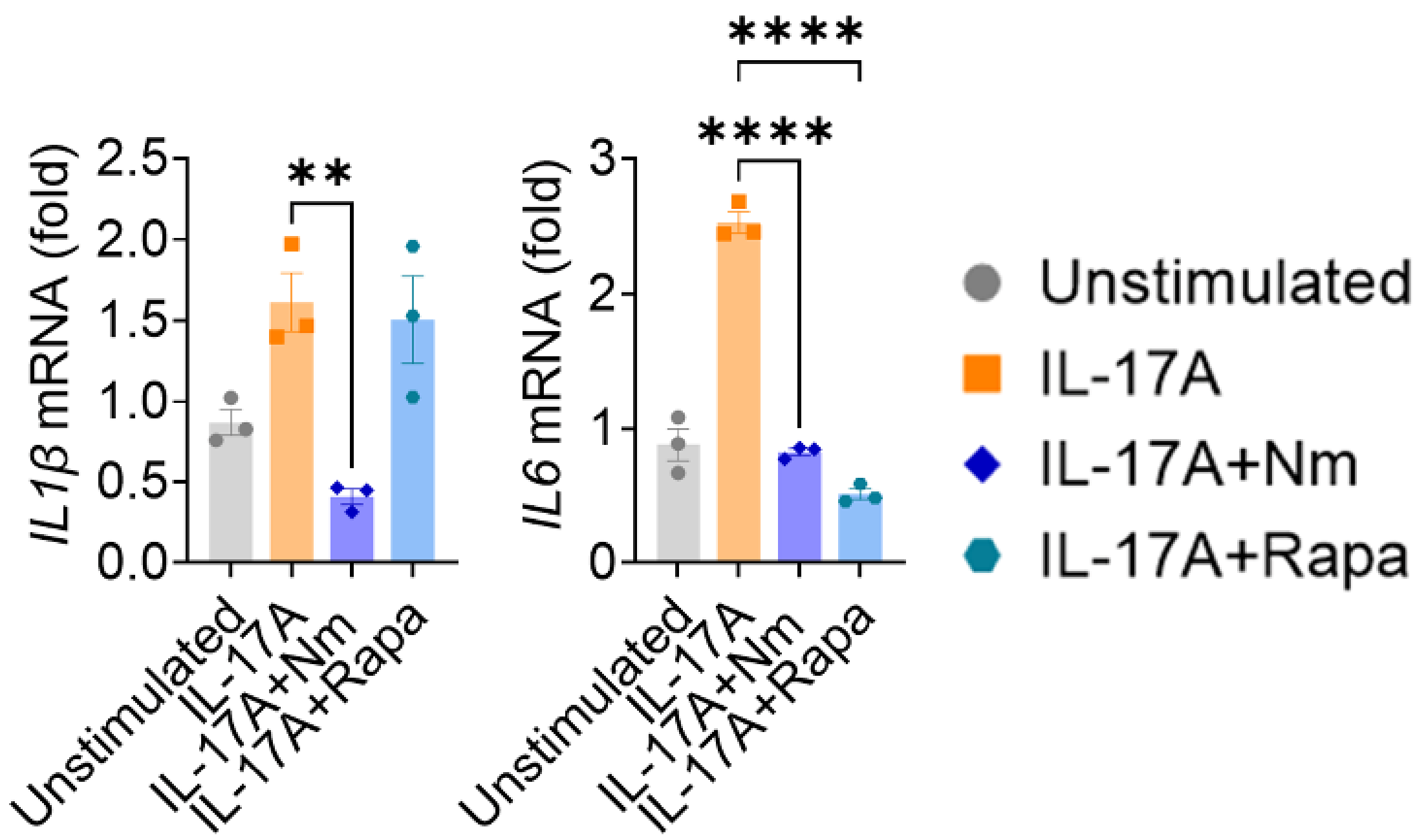

Supplementary Figure S4. Nomilin reduces IL-17A-induced expression of inflammatory genes in primary human keratinocytes. Primary human keratinocytes were stimulated with IL-17A in the presence or absence of nomilin or rapamycin. mRNA expression of pro-inflammatory cytokine genes (*IL1β* and *IL6*) was analyzed by qPCR and expressed as fold change relative to unstimulated control. Data are presented as mean ± SEM from independent experiments. Statistical significance was determined by one-way ANOVA followed by Holm-Šídák multiple comparison test. ****P < 0.0001, **P < 0.01.

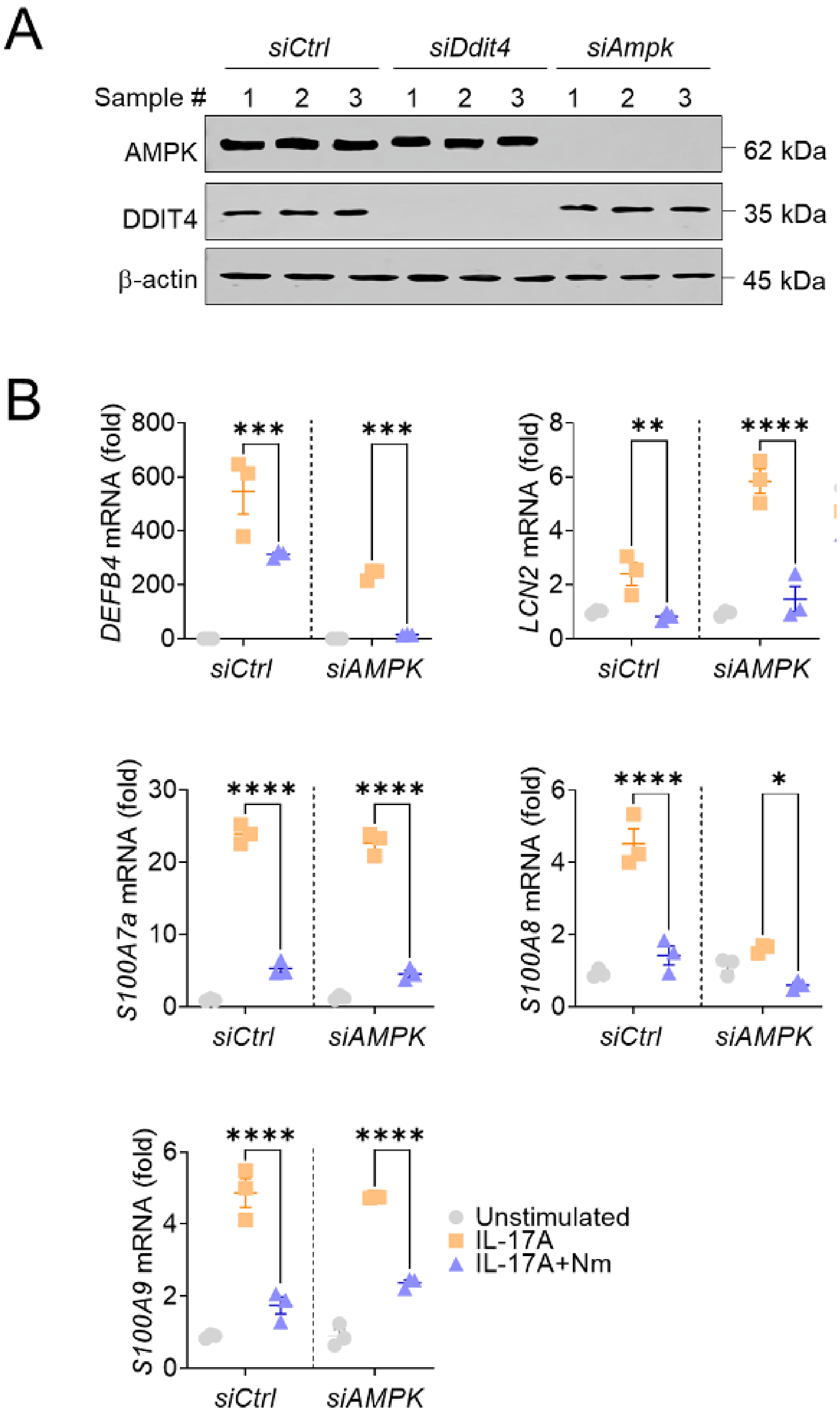

A
siCtrl
siDdit4
siAmpk
Sample #
1
2
3
1
2
3
1
2
3
AMPK
DDIT4
β-actin
62 kDa
35 kDa
45 kDa
B
DEFB4 mRNA (fold)
LCN2 mRNA (fold)
S100A7a mRNA (fold)
S100A8 mRNA (fold)
S100A9 mRNA (fold)
siCtrl
siAMPK
Unstimulated
IL-17A
IL-17A+Nm

Supplementary Figure S5. Validation of DDIT4 silencing and assessment of AMPK knockdown in primary human keratinocytes. Primary human keratinocytes were transfected with siRNA targeting DDIT4 or AMPK prior to IL-17A stimulation. **(A)** Representative Western blotting images showing reduced DDIT4 and AMPK protein levels following siRNA transfection. **(B)** mRNA expression of inflammatory genes following AMPK knockdown and nomilin treatment, analyzed by qPCR. Data are presented as mean ± SEM from independent experiments. Statistical significance was determined by one-way ANOVA followed by Holm-Šídák multiple comparison test. ****P < 0.0001, ***P < 0.001, **P < 0.01, *P < 0.05.

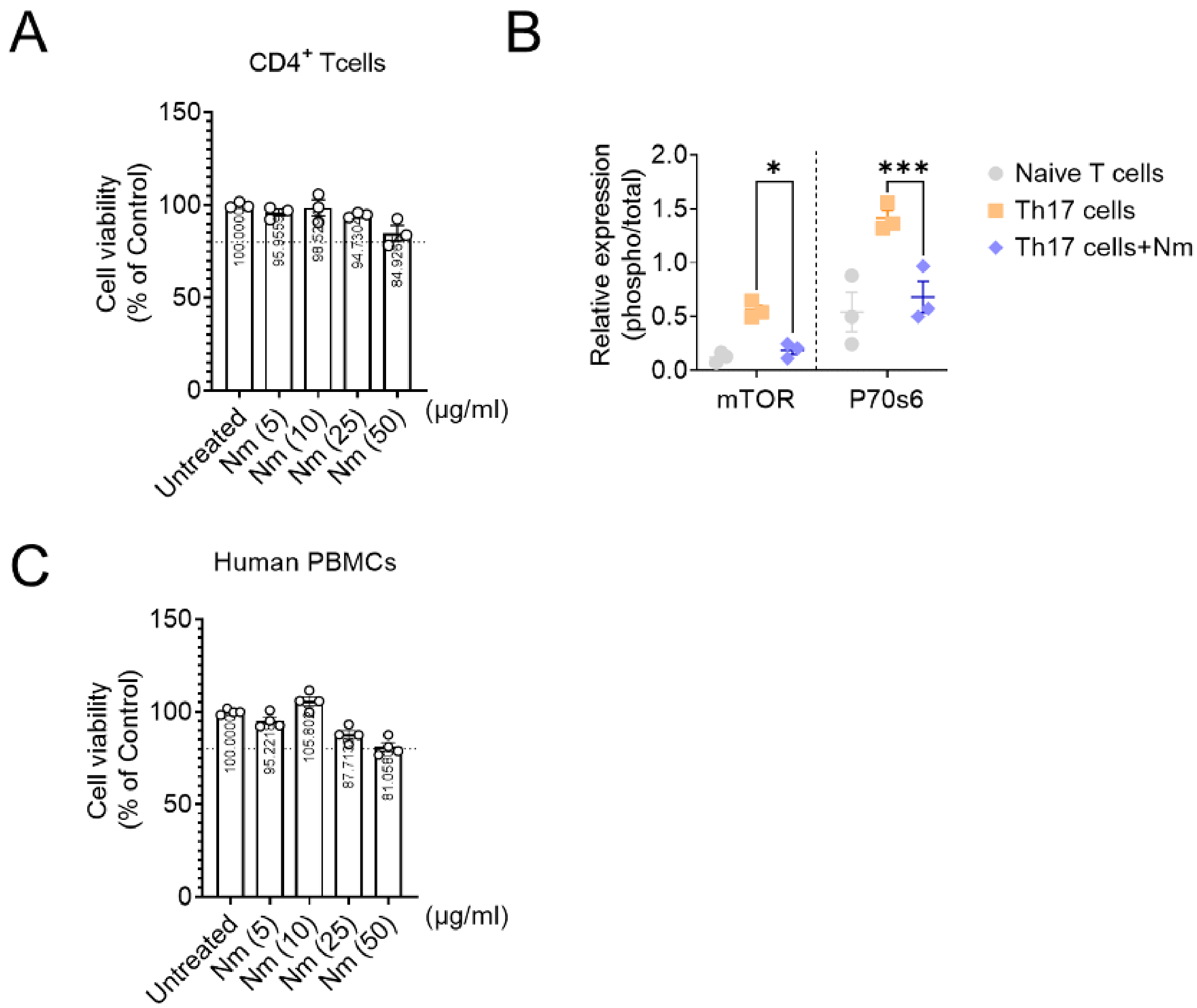

Supplementary Figure S6. Effects of nomilin on cell viability and mTOR signaling in T cells and PBMCs. (A) Cell viability of mouse CD4+ T cells treated with the indicated concentrations of nomilin for 48 h, assessed by CCK-8 assay. **(B)** Densitometric quantification of phosphorylated mTOR and p70S6K shown in Figure 7C, normalized to total protein levels. **(C)** Cell viability of human PBMCs treated with the indicated concentrations of nomilin for 72 h, assessed by CCK-8 assay. Data are presented as mean ± SEM from independent experiments. Statistical significance was determined by one-way ANOVA followed by Holm-Šídák multiple-comparison test. ***P < 0.001, *P < 0.05.

References

1. Hiraganahalli Bhaskarmurthy D, Evan Prince S. Effect of Baricitinib on TPA-induced psoriasis like skin inflammation. Life Sci. 2021; 279: 119655.
2. Park HS, Sung WJ, Park YY, Hong J, Oh HK, Lee HS. Sesamin Induces MCL-1-Dependent Apoptosis in Activated T Cells and Ameliorates Experimental Atopic Dermatitis. Int J Biol Sci. 2025; 21: 4719-35.
